# Naturally Occurring 8ß,13ß-kaur-15-en-17-al and Anti-Malarial Activity from *Podocarpus polystachyus* Leaves

**DOI:** 10.3390/ph15070902

**Published:** 2022-07-21

**Authors:** Mira Syahfriena Amir Rawa, Mohammad G. Al-Thiabat, Toshihiko Nogawa, Yushi Futamura, Akiko Okano, Habibah A. Wahab

**Affiliations:** 1 School of Pharmaceutical Sciences, Universiti Sains Malaysia, Minden 11800, Malaysia; mirasyah@usm.my (M.S.A.R.); mohd.althiabat@gmail.com (M.G.A.-T.); 2 Collaborative Laboratory of Herbal Standardization (CHEST), School of Pharmaceutical Sciences, Universiti Sains Malaysia, Bayan Lepas 11900, Malaysia; 3 USM-RIKEN Interdisciplinary Collaboration for Advanced Sciences (URICAS), Universiti Sains Malaysia, Minden 11800, Malaysia; nogawat@riken.jp; 4 Molecular Structure Characterization Unit, Technology Platform Division, RIKEN Center for Sustainable Resource Science, 2-1 Hirosawa, Saitama 351-0198, Japan; 5 Chemical Biology Research Group, RIKEN Center for Sustainable Resource Science, 2-1 Hirosawa, Wako, Saitama 351-0198, Japan; futamuray@riken.jp (Y.F.); aokano@riken.jp (A.O.)

**Keywords:** *Podocarpus polystachyus*, Podocarpaceae, kaurene, anti-malarial, *Plasmodium falciparum*, *Pf*LDH, isolation, structure elucidation, molecular docking, molecular dynamics

## Abstract

Despite much interest and studies toward the genus *Podocarpus*, the anti-malarial evaluation of *Podocarpus polystachyus*’s phytoconstituents remains lacking. Herein, the phytoconstituents of *P. polystachyus* leaves and their anti-malarial effect against *Plasmodium falciparum* were investigated for the first time. One new natural product, 8ß,13ß-kaur-15-en-17-al (**1**), along with three known compounds, 8ß,13ß-kaur-15-en-17-ol (**2**) and 13ß-kaur-16-ene (**3**), and α-tocopherol hydroquinone (**4**) were isolated via HR-ESI-MS and NMR analyses. Compounds **1** and **2** inhibited *P. falciparum* growth at 12 and 52 µM of IC_50_, respectively. Their anti-malarial activity was associated with the in silico *P. falciparum* lactate dehydrogenase (*Pf*LDH) inhibition. Molecular docking of ligands **1** and **2** with the putative target *Pf*LDH revealed ~−2 kcal/mol of binding energies more negative than the control. Molecular dynamic simulations (100 ns) showed equal or smaller deviation values (RMSD, RMSF, Rg) and stronger interactions of *Pf*LDH-**1** and *Pf*LDH-**2** complexes via at least one consistent H-bond than the control. Additionally, a slightly increased *Pf*LDH H-bond profile in their interactions improved the *Pf*LDH dynamic and structural stabilities. Overall, this study supports the relevance of **1** and **2** as plasmodial growth inhibitors with their putative anti-*Pf*LDH activity, which could be a potential scaffold for developing anti-malarial drugs.

## 1. Introduction

*Podocarpus* is an ancient gymnosperm genus belonging to Podocarpaceae, the second biggest family of conifers [1]. It is widely distributed in New Zealand, Australia, and Asia continents including China and Japan [2]. The phytoconstituents of *Podocarpus* have been extensively reported since the early 1900s. They primarily constitute diterpenoids and bioflavonoids such as amentoflavanone and hinokiflavanone, which are important taxonomical markers in differentiating the new and old taxa [3]. Norditerpene dilactones including nor- and bis-diterpenes represent the most prevalent secondary metabolites in the genus. These bioactive components are responsible for various biological activities such as anti-tumor [4], anti-viral [5], anti-inflammatory [6], and diuretic [7] agents.

*P. polystachyus* R. Br. ex Endl. is native to Singapore, the Philippines, Thailand, the Moluccas, New Guinea, Indonesia, and Malaysia [8]. The tree can grow up to 20 m tall. Despite various medicinal properties reported from the genus, *P. polystachyus* is limitedly used to treat rheumatic and painful joints [8]. It promotes phytoremediation that can improve diesel fuel-contaminated soils [9,10]. Its fruit is edible, and its application is primarily focused on timber and products to make softwood and furniture. The wood extract of *P. polystachyus* consists of phenolic diterpenoids such as totarol, 19-oxototarol, 4b-carboxy-19-nortotarol, and macrophyllic acid [11]. 

Malaria is a major infectious disease that affects millions of people worldwide, causing death mostly in young children in sub-Saharan Africa [12]. It is caused by the protozoan parasites *Plasmodium* [13], with *Plasmodium falciparum* being the most virulent form of the malarial parasite. The transmission occurs in humans through the bite of *Anopheles* mosquitoes carrying the parasite [13]. Artemisinin is one of the effective anti-malarial drugs exerting broad activities against all parasite phases found in human erythrocytes. A drug cocktail regimen involving artemisinin is still encouraged, but the rise of drug resistance in certain countries demands a new drug combination therapy [14]. Most anti-malarial drugs have been exercised for more than a decade. Furthermore, the gradual rise in *P. falciparum* malaria drug resistance has put researchers to the task of developing more effective drugs, especially against *P. falciparum* targets [14].

The strategies of developing anti-malarial drugs take into account the metabolic differences between the malarial parasite and its host, for example, nucleic acid metabolism, haem detoxification, oxidative stress, and fatty acid biosynthesis [15]. Proteins involved in the metabolic pathways such as plasmepsin I, plasmepsin II, plasmepsin V, falcipain 2, pyridoxal kinase, and *P. falciparum* lactate dehydrogenase (*Pf*LDH) are among the reported anti-malarial drug targets [15,16]. *Pf*LDH is an essential enzyme that mediates the parasite’s anaerobic glycolysis. In the presence of NADH, it reduces pyruvate into lactate to produce adenosine triphosphate (ATP) production, which is crucial for the parasite’s survival [16,17]. It is distinguished from other LDHs including human LDH due to its unique amino acids at its active site [16]. Moreover, it is highly expressed during the blood-stage that is directly correlated to the parasitemia level, which has put *Pf*LDH as an effective target for the structure-based design of new anti-malarial drugs.

**Figure 1:**
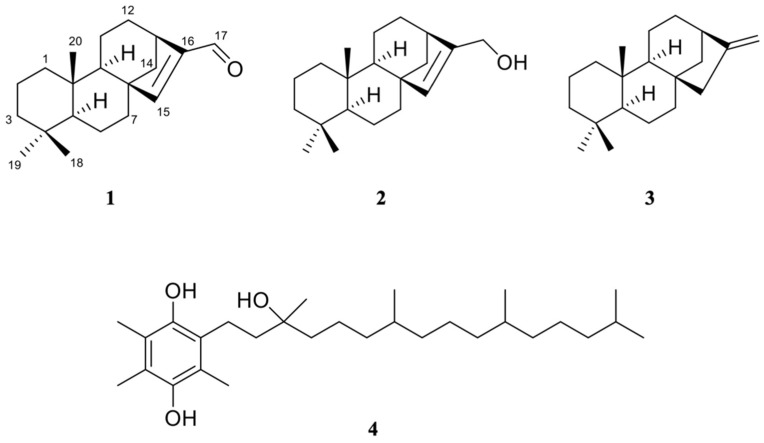
Structures of **1**–**4**.

*Podocarpus* species were predominantly investigated for their anti-proliferative studies, owing to their diverse and unique diterpenoids. For example, hydroxymakilactone derivatives exhibited potent inhibitory activities against tumor cell lines (HeLa, HepG-2, AGS, MDA-MB-231, and PANC-1) ranging from 0.3–27 µM of IC_50_ [4]. The potential of *Podocarpus* species exhibiting anti-malarial effect, however, remains lacking and has yet to be explored further. So far, an abietane-type diterpenoid found in *Podocarpus ferruginea* named ferruginol was demonstrated to exert potent anti-plasmodial activity against *P. falciparum* 3D7 and K1 strains at nanomolar concentrations [18]. As limited studies were made on *P. polystachyus*, this research aimed to perform a phytochemical study and evaluate the anti-malarial activity of this species against the *P. falciparum* 3D7 strain. Isolation of *P. polystachyus* afforded three kaurene derivatives including compound **1** as a new natural product, and one vitamin E derivative (Figure 1). The plasmodial growth inhibition by the kaurene derivatives was correlated with the in silico anti-*Pf*LDH activity via computational analyses to gain a better insight into their molecular mechanism. Molecular docking and molecular dynamics (MD) simulations predicted the binding affinity, stability of the complexes, dynamic expression, and free binding energy of the kaurene derivatives when in a complex with *Pf*LDH.

## 2. Results and Discussion

### 2.1. Isolation and Identification of Chemical Constituents

Seven grams of P. polystachyus hexane extract from leaves was subjected to medium pressure liquid chromatography (MPLC) on a SiO_2_ column to afford eight fractions (Figure S1). The fourth and fifth fractions each showed a major peak with a UV characteristic of 250*–*260 nm in the LC chromatogram. This UV characteristic was hypothesized to represent a diterpenoid and therefore was further investigated. The fourth fraction was separated by C_18_-MPLC to obtain two major sub-fractions. C_18_-HPLC purification was conducted for the first sub-fraction that afforded compound **1** (0.6 mg). The second sub-fraction was purified by C_18_-HPLC to afford compound **4** (30.0 mg). The fifth fraction was separated by C_18_-MPLC and purified by C_18_-HPLC to obtain 2.4 mg of compound **2**. Additionally, a compound peak with a similar UV characteristic of 250*–*260 nm was observed in the low polar fraction of the EtOAc extract after separation by SiO_2_-MPLC. Further separation of this fraction afforded crude compound **3** which was purified by C_18_-HPLC to obtain 6.1 mg of **3**. 

The molecular formula of **1** was determined to be C_20_H_30_O by high-resolution ESI-TOF-MS (found m/z 287.2365 [M+H]^+^ for C_20_H_31_O 287.2369 and 269.2258 [M+H-H_2_O]^+^ for C_20_H_29_ 269.2264) (Figure S2). The molecular formula of **2** was determined to be C_20_H_32_O by the interpretation of high-resolution ESI-TOF-MS, which did not show [M+H]^+^ ion but showed [M+H-H_2_O]^+^ and [2M+H-H_2_O]^+^ ions (found m/z 271.2417 [M+H–H_2_O]^+^ for C_20_H_31_ 271.2420 and 559.4877 [2M+H-H_2_O]^+^ for C_40_H_63_O 559.4873) (Figure S3), in comparison to that of **1**. The molecular formula of **3** was determined to be C_20_H_32_ by high-resolution EI-TOF-MS (found m/z 272.2503 [M]^+^ for C_20_H_32_ 272.2504) (Figure S4). The molecular formula of **4** was determined to be C_29_H_50_O_3_ by high-resolution ESI-TOF-MS (found m/z 447.3832 [M+H]^+^ for C_29_H_51_O_3_ 447.3833 and 429.3727 [M+H–H_2_O]^+^ for C_29_H_49_O_2_ 429.3727) (Figure S5). Based on the spectral features (Figures S16–S29), compounds **2** and **3** were identified as 8ß,13ß-kaur-15-en-17-ol (17-isophyllocladenol) [19] and 13ß-kaur-16-ene (phyllocladene) [20], respectively. 

The ^13^C NMR spectrum of **4** denoted 29 signals (Figure S31). Its DEPT experiment results showed eight methyls, 11 methylenes, three methines, a quaternary signal at 74.7 ppm attributable to a hydroxyl sp^3^ carbon, and six quaternary signals above 115 ppm representing a phenyl ring (Figure S32). The 2D NMR spectra of **4** suggested the presence of an alkyl chain with four methyl groups attached to three methine carbons (Figures S33–S35). Based on the comparison of these NMR characteristics with reported data [21], **4** was identified as α-tocopherol hydroquinone, a vitamin E derivative. Two carbons observed at 144–146 ppm constituting OH alkenes justified that the hydroquinone moiety was oxidized to quinone during the ESI process of **4**. This type of oxidation is commonly observed for a phenolic hydroxyl group in the ESI process and explains the loss of two protons. 

Compound **1** was isolated as a white amorphous powder. The ^1^H NMR spectrum of **1** showed three singlet methyl signals between 0.73–0.86 ppm, overlapping signals between 1.10–1.73 ppm attributable to methylene protons, and a doublet of doublets proton signal integrating 1H at 1.82 ppm (Figure S6). Two singlet protons (6.85 and 9.71 ppm) observed at the lower field implied the presence of alkenyl hydrogen and an aldehyde. The ^13^C NMR spectrum of **1** suggested 20 signals, which was in agreement with the structure of a diterpene (Figure S7). Four quaternary carbons including one sp^2^ carbon at 147.6 ppm were observed. The carbon chemical shifts seen at 157.5 and 190.1 ppm agreed with the ^1^H NMR that indicated the presence of a hydrogenated alkene and an aldehyde. 

The 2D NMR data of **1** revealed a correlation of a tetracyclic diterpene with a cyclopentene unit that fused with the main skeleton, a perhydrophenantrene unit, which connected at C-8 and C-13 (Figures S9–S12). The HMBC and DQF-COSY correlations between the aldehyde and alkene confirmed the structure was a diterpene aldehyde. The previous isolation of a diterpene aldehyde from Cryptomeria japonica, ent-kaur-15-en-17-al, manifested 5R*, 8S*, 9S*, 10R*, 13R* relative configuration [22]. Its ^13^C NMR showed a few carbon chemical shift disagreements with **1**, notably its C-9 and C-14 had chemical shifts of 46.8 and 43.0 ppm, respectively (Table 1). Ours were observed at lower-fielded chemical shifts of 54.20 and 54.17 ppm for C-9 and C-14, respectively. The NOESY cross-peaks/correlations of H-15 (δH 6.85) with H-20 (δH 0.73), H-6 (δH 1.40) and H-7 (δH 1.40) indicated that ***1*** has an 8ß,13ß-orientation with a relative configuration of 5S*, 8S*, 9R*, 10S*, 13R*, not 5R*, 9S*, 10R* (Figure S12). 

The relative structure was verified by the comparison of ^13^C NMR chemical shifts between experimental and calculated data. The calculation was carried out for 8ß,13ß-kaur-15-en-17-al forming 5S, 8S, 9R, 10S, 13R conformer and chemical shifts were obtained by DFT at ωB97XD/6-31G* level based on the optimized conformers by DFT at ωB97XD/6-31G* level (Table 1). The result was in agreement with the experimental NMR (Figure S13), confirming **1** was 8ß,13ß-kaur-15-en-17-al, a newly reported natural product. The absolute configuration was confirmed by the comparison of the ECD spectrum to the theoretical ones for both the 5S-isomer and its enantiomer, which were simulated by TD-DFT at CAM-B3LYP/6-31+G(d) level based on the optimized conformers by the same theory used for NMR calculation (Figure S14). The resulting simulated ECD spectrum of the 5S-isomer showed a good agreement with the experimental ECD spectrum and was in contrast to that of the enantiomer, suggesting that **1** had the 5S, 8S, 9R, 10S, and 13R configurations (Figure S15).

Compound **1** was chemically synthesized from **3** with selenium dioxide through oxidation in acetic acid heating [23]. Fujita and Ochiai [24] demonstrated successful conversion of the alcohol moiety of **2** with thallium trinitrate (TTN) to an αß-unsaturated aldehyde in methanol. It is noteworthy that ent-kaurene is a diastereomer of **3** [25], and its oxidation reaction produces ent-kaur-15-en-17-ol and ent-kaur-15-en-17-al [24]. The similar stereochemistry of **2** and **3** provide evidence for **1** to be involved in the similar biosynthetic pathway. Podocarpacane, abietane, totarane, and kaurane derivatives are among the major diterpenoid groups reported in Podocarpus species [26].

### 2.2. In Vitro Anti-Malarial Activity

In vitro anti-malarial results revealed that **1** fairly inhibited *P. falciparum* growth at 12 µM of IC_50_, followed by **2** at 52 µM of IC_50_. Compounds **3** and **4** showed no significant activity. The presence of the perhydrophenantrene unit was deemed to have modified the activity strength as **3** showed no inhibition. Oxidation of alcohol to aldehyde increased the activity strength as observed between **2** and **1**, but such notion warrants further structure–activity relationship study.

Chemically synthesized and natural ent-kaurene derivatives isolated from Espelletiinae species demonstrated anti-malarial activity against *Plasmodium berghei* infected in male albino mice [27]. Another report showed good anti-malarial activity from chemically synthesized oxidized/epoxidized *ent*-kaurane derivatives against the W2 strain of *P. falciparum* [28]. To our best knowledge, besides ferruginol [12], this study has added another pharmacological value to naturally occurring compounds of *Podocarpus* species against malaria.

### 2.3. In Silico Anti-Malarial Activity

#### 2.3.1. Molecular Docking Analysis

*Pf*LDH plays a key role in the glycolytic pathway of *P. falciparum* [16]. It is essential for the parasite’s survival to produce energy via anaerobic glycolysis by reducing pyruvate into lactate [16,17]. As *Plasmodium* highly depends on the anaerobic glycolysis for energy generation, targeting PfLDH in chemotherapy can impede ATP production and cause immediate cell death [16,17]. Additionally, *Pf*LDH is genetically unique and different from the human LDH, making *Pf*LDH one of the viable malaria enzyme targets [16]. Several previous studies have estimated the molecular mechanism of bioactive compounds via computational analyses, incorporating *Pf*LDH as a putative target [29,30,31]. Similarly, in this in silico study, *Pf*LDH served as a putative enzyme target to predict the anti-malarial effect of hybrid molecules **1** and **2**.

To validate the docking procedure, the co-crystallized ligand 3,5-dihydroxy-2-naphthoic acid (control) was extracted from the crystal structure (PDB code: 1U5A) [32] and re-docked into the same active binding site of the *Pf*LDH via AutoDock 4.2 (Figure S36). The score showed that the method was able to pose the control in almost the same conformation as its crystal structure, with an RMSD of ~2.0 Å and a binding affinity of ~−5.94 kcal/mol (Table 2). The ability of AutoDock 4.2 to repose the co-crystallized structure and reserve the key interactions with ARG171 (guanidinium side chain), HIS195, and ALA236 is remarkable as ARG171 is known to be highly conserved in the active site [33].

**Figure 2:**
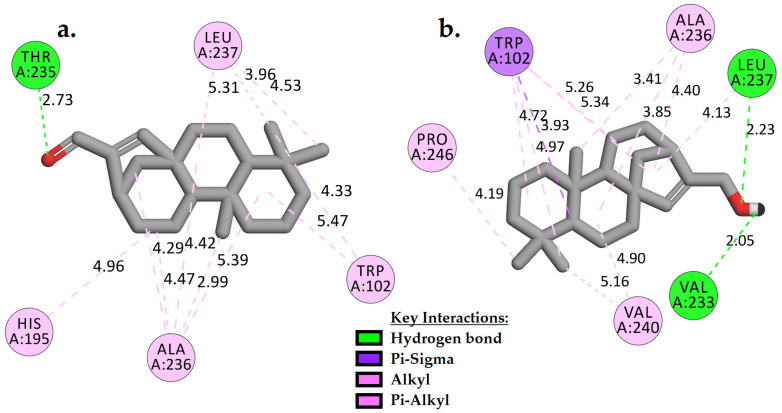
Two-dimensional interaction analysis of docked models of compounds **1** (**a**) and **2** (**b**) with *Pf*LDH binding site.

Compounds **1** and **2** showed about −2 kcal/mol of binding energies stronger (more negative) than the control (Table 2). The lower the binding energy, the more stable the complex is. Based on Figure 2, it is clear that **1** and **2** positioned themselves in the hydrophobic groove of the active site by forming multiple hydrophobic contacts with the adjacent residues such as TRP102, ALA236, LEU237, and/or HIS195 and VAL240. Additionally, the carbonyl group in **1** (Figure 2a) formed a moderate strength of H-bond with THR235 (2.73 Å). As observed in **2**, two slightly stronger H-bonds were created with VAL233 and LEU237, at distances of 2.05 Å and 2.23 Å, respectively, when the carbonyl group was replaced by a hydroxyl side chain (Figure 2b).

#### 2.3.2. Molecular Dynamics (MD) Analysis

The molecular dynamics analysis explored the binding stability of compounds **1** and **2** in complex with *Pf*LDH and their complex’s dynamical behavior. As both tested compounds showed more negative binding energies (~−2 kcal/mol) than the control, they were further subjected to 100 ns MD simulations. Additionally, the enzyme crystal structure (PDB ID: 1U5A) in complex with the control was also simulated for comparison. The behavior of each complex system was compared to that of the ligand-free system (apo-*Pf*LDH). Prior to the simulation, 3,5-dihydroxy-2-naphthoic acid was removed from apo-*Pf*LDH to serve as a starting structure for the simulation [32]. 

**Figure 3:**
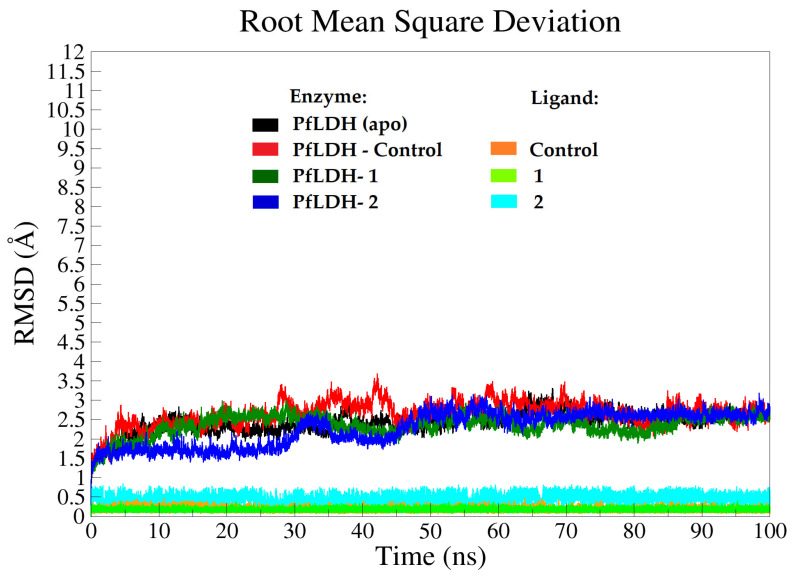
The root mean square deviation (RMSD) plots of the enzyme and ligand backbone atoms for the selected systems. Apo-*Pf*LDH (black), *Pf*LDH-control (red and orange), *Pf*LDH-1 (Dark green and lime), and *Pf*LDH-2 (blue and cyan).

Root-mean-square deviation (RMSD) of the enzyme backbone and ligand atoms was tracked throughout the MD simulations (100 ns) to determine the stability of the simulated systems. In general, stable RMSD values ranging from 1.8–3.5 Å were observed in all systems (enzyme backbones) during the simulation time, reaching equilibrium after 45 ns (Figure 3). The initial and average structures after MD simulations were compared. A stable RMSD value of ~2.5 Å was observed in the apo form (black plot), reaching equilibrium after 18 ns with almost similar fluctuations on average until 100 ns (Figure 3). This observation is closely in agreement with the results demonstrated by Saxena et al. [15]. In the *Pf*LDH-control system, the average RMSD of the *Pf*LDH backbone was seen between 2.4 Å and 3.5 Å. It increased from 2.4 Å to 3.4 Å at 27 ns, then continued to fluctuate after 83 ns until 100 ns of simulation between 2.1 Å and 3.4 Å. The deviation was less than 2 Å. 

The enzyme backbone in the *Pf*LDH-**1** system reached its equilibrium after 20 ns, showing steady RMSD values of 2.0–3.0 Å. It is also noteworthy that the RMSD value of *Pf*LDH in this system was lower than that of *Pf*LDH in the *Pf*LDH–control system, indicating higher stability of *Pf*LDH with **1** throughout the simulations. On the other hand, the RMSD value of *Pf*LDH-**2** showed that the enzyme backbone followed two distinct phases, but the difference did not significantly affect the stability of the complex. The first phase can be seen from 5 to 30 ns with an RMSD value of ~1.8 Å, and the second phase from 50 to 100 ns with an average RMSD of 2.6 Å (Figure 3). It is worth noting that the complex systems with **1** and **2**, in general, showed slightly lower RMSD values than apo and halo (bounded to control), which may indicate that the enzyme is more dynamically stable with these compounds.

**Figure 4:**
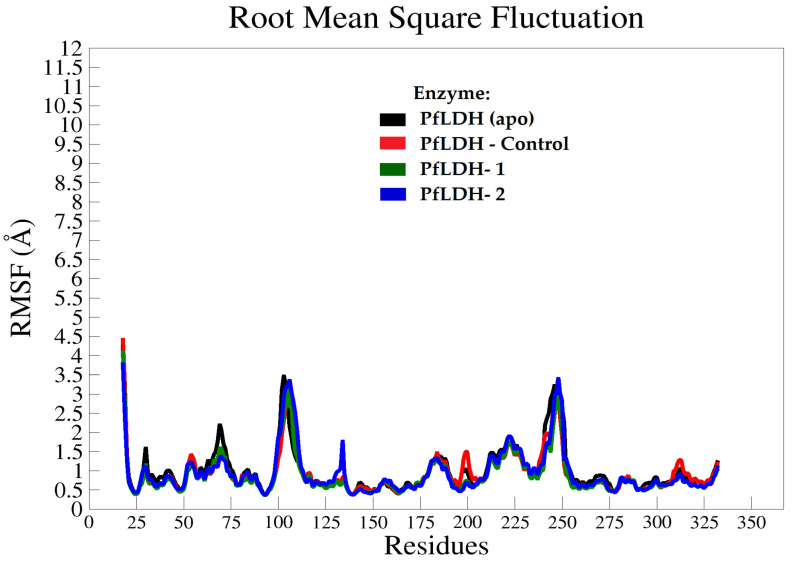
The RMSF graphs of the *Pf*LDH backbone atoms throughout the 100 ns MD simulation time for all systems. The RMSF values indicate the residual atomic fluctuations of each amino acid residue when they interact with the ligands throughout the trajectory.

Root mean square fluctuation (RMSF) was measured in all systems throughout 100 ns of the MD simulation time, which corresponded to the structural flexibility of the *Pf*LDH backbone atoms. The average RMSF values were observed to be less than 3.5 Å for all systems of the *Pf*LDH backbone atoms (except for terminal residues) (Figure 4), with no significant differences in the RMSF values between apo and halo systems. This clearly shows that the structural stability of *Pf*LDH can be achieved even in the presence of lead molecule inhibitors such as compounds **1** and **2**.

**Figure 5:**
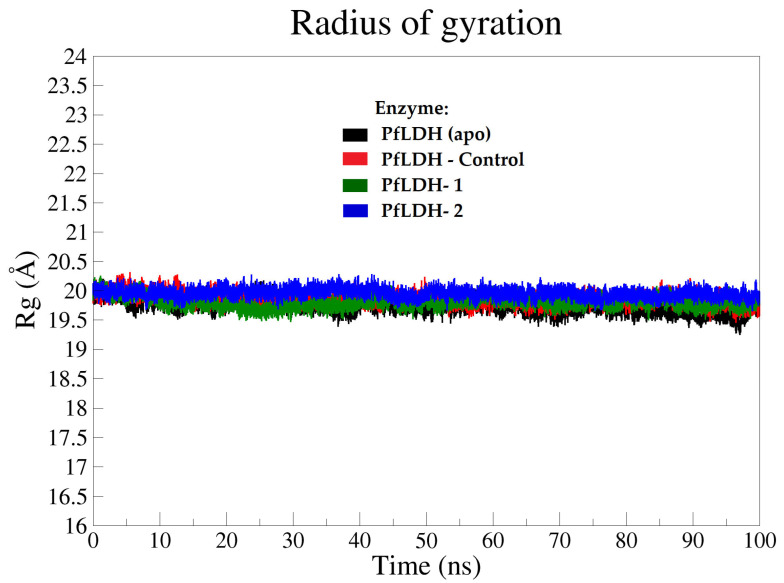
Radius of gyration (Rg) plots of the *Pf*LDH backbone atoms of all systems at MD interval time (0–100 ns); Apo-*Pf*LDH (black), *Pf*LDH-control (red), *Pf*LDH-**1** (dark green), and *Pf*LDH-**2** (blue).

The compactness and the size of the enzyme molecules in apo and halo forms were determined based on the calculations of the radius of gyration (Rg) throughout the MD simulation time of 0–100 ns [34]. The Rg plot results illustrated that all four systems were stable, with a consistent value of approximately 19.9 Å (Figure 5).

Due to the consistency and stability at region 90–100 ns shown by the RMSD (Figure 2) and simulation time plots, the obtained coordinates from the production phase of this region were used to calculate the free binding energies of the simulated systems. The free binding energies with a neglected entropic contribution of the simulated systems were determined based on the Molecular Mechanics-Poisson Boltzmann surface area (MM-PBSA) program [35] implemented in AMBER 18. This method can calculate the difference in free energy between two states, which are usually the bound and unbound states of two solvated molecules, as well as compare the free energy of two solvated conformations of the same molecule [36]. 

The trend in the ligand–enzyme interactions observed in the post-MD simulations was in contrast with that observed in the molecular docking result. The docking scores ranked the binding free energies (more negative) as 1 = 2 > control. The MM-PBSA scores, on the other hand, ranked the binding free energies as 1 > 2 > control. This may justify the in vitro anti-malarial activity, where **1** showed more potent inhibition than **2**. The MM-PBSA method is considered to be more computationally accurate than the docking scoring functions (empirical or knowledge-based scoring functions) as it can detect conformational changes caused by ligand binding and allow for rigorous free energy decomposition into contributions from different groups of atoms or types of interactions [37,38]. 

Table 3 showed favorable binding free energies of *Pf*LDH–**1**, *Pf*LDH–**2**, and *Pf*LDH–control (−30.87, −24.70, and −16.64 kcal/mol, respectively). Both compounds **1** and **2** formed stronger interactions with *Pf*LDH based on their higher binding free energies than the control, with electrostatic and van der Waals interactions being the major contributors, along with the polar solvation energy term. The hydrophobic interactions are believed to contribute to the most important aspect of their interactions in the *Pf*LDH pocket. Based on the results, all three ligands expressed almost the same values of non-polar solvation energy, while a significant difference in their polar solvation energy was observed. This observation could be related to the higher number of H-bonds (3–4 bonds) formed by *Pf*LDH-control in comparison to H-bonds (1–2 hydrogen bonds) formed by *Pf*LDH-**1** and *Pf*LDH-**2** complexes (Figure S37). The difference in this energy has affected the overall binding free energy observed in **1** with almost −14 kcal/mol better (more negative) than the control.

**Figure 6:**
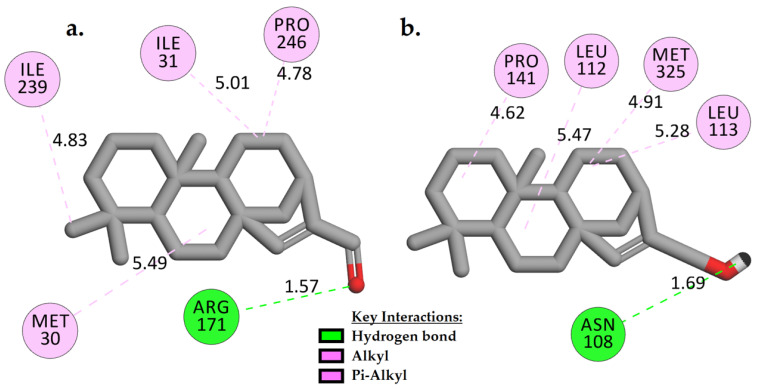
Two-dimensional interaction models show how H-bonds are formed between *Pf*LDH-**1** (**a**) and *Pf*LDH-**2** (**b**) with the amino acid residues in the *Pf*LDH active binding site at 100 ns (last snapshot) of MD time intervals.

H-bond is one of the critical interactions required for any efficient enzyme–ligand complex formation, as it retains the molecule tightly in the active site of the enzyme [15]. The greater the number of intermolecular hydrogen bonding interactions, the more stable the complex is. The H-bond profiles of the interacting ligands in the binding site were investigated in this study (Figure S37) to observe the effect of the ligands on their mechanism of binding with *Pf*LDH throughout the MD simulation time (100 ns) [15,39]. The number of H-bonds created within the enzyme itself and with ligands was analyzed (Figure S37). In the *Pf*LDH (apo form) system, the H-bond profile of the enzyme structure revealed consistent residue interactions throughout the MD simulation time with an average of ~83 bonds (Figure S37a). The *Pf*LDH-control system showed a similar H-bond profile, averaging at ~83 bonds (Figure S37b). On the other hand, a slight increase in the H-bond profile of the *Pf*LDH-**1** and *Pf*LDH-**2** systems (average of 84 bonds) was observed (Figures S37c and S37d), suggesting compounds **1** and **2** may improve the structural stability of *Pf*LDH. Interestingly, **1** and **2** formed at least one consistent H-bond with the active binding site residues (Figure 6). They showed higher binding affinity than the control in the MM-PBSA experiment, probably due to the van der Waals interactions observed as the primary contributors (Table 3).

Target identification via in silico approaches has become increasingly popular in the development of novel drugs. Numerous studies have suggested that the *Pf*LDH enzyme is an effective target for developing novel inhibitors [15,16]. In this study, we found that compounds **1** and **2** efficiently docked into the active site of *Pf*LDH with minimum free binding energy. Thus, it is feasible to predict the molecular mechanism of these compounds with *Pf*LDH via the in silico docking study. The 100 ns molecular dynamic simulation (RMSD, RMSF, Rg) revealed that the observed deviation values of docked complexes were lesser or equal to the range of the reference protein, i.e., the first frame of the simulation. This indicates that the complexes were stable during the molecular dynamic simulation, with no significant conformational changes observed.

The binding interactions of an inhibitor with an enzyme molecule often lead to changes in the secondary structure. Monitoring the variation in the secondary structure as a function of simulation time is crucial to ascertain the formation of a stable enzyme–inhibitor complex. It can be concluded that compounds **1** and **2** behaved well within the active site of the *Pf*LDH enzyme. The MM-PBSA experiment confirmed **1** and **2** formed interactions with the *Pf*LDH active site via H-bonds and van der Waals interactions with other residues. In fact, compounds **1** and **2** were able to retain their binding interactions within the active site residues during the post-simulation, validating the stability of **1** and **2** as putative *Pf*LDH inhibitors when in complex with the enzyme.

## 3. Materials and Methods

### 3.1. Instrument

LC-MS analysis was performed on Waters UPLC-H-Class system (Waters, Milford, MA, USA) connected to AB Sciex API 3200 by ESI probe (AB Sciex, Framingham, MA, USA) on a Waters BEH C18 column (2.1 mm i.d. × 50 mm, 1.7 μm) with elution of acetonitrile/0.05% aqueous formic acid linear gradient system (acetonitrile: 5 to 100% in 4 min at 0.5 mL min^−1^). HR-ESI-TOF-MS was measured by Waters VION IMS QTof system. JEOL JMS-T100GCV was used for HR-EI-TOF-MS analysis (JEOL, Tokyo, Japan). Teledyne ISCO CombiFlash Companion (Teledyne ISCO, Lincoln, NE, USA), and RediSep Rf Gold silica column or RediSep Rf Gold HP C18 were used for MPLC. Preparative HPLC was performed using Waters 600E pump system with Senshu Pak Pegasil ODS column (20 mm i.d. × 250 mm or 10 mm i.d. × 250 mm, 5 μm). The NMR data were obtained at 500 MHz for ^1^H NMR and 125 MHz for ^13^C NMR on JEOL JNM-ECA-500 spectrometer (JEOL, Tokyo, Japan). Chemical shifts (in ppm) were referenced based on the residual undeuterated solvent. 

### 3.2. Plant Material

*Podocarpus polystachyus* R. Br. ex Endl. was collected at Universiti Sains Malaysia (USM), Pulau Pinang. The sample was prepared by Mr. Muhammad Hilmi Jamaluddin and authenticated by Dr. Farah Alia Nordin. The specimen ID no. 11875 was deposited at the USM Herbarium, Institute of Biological Sciences, USM, Pulau Pinang (Figure S38). The plant name was checked and confirmed from the Plant List (www.theplantlist.org). This study followed the IUCN Policy on Research Involving Species at Risk of Extinction and the Convention on the Trade in Endangered Species of Wild Fauna and Flora.

### 3.3. Extraction and Isolation

The plant materials of *P. polystachyus* leaves (113 g) were extracted with methanol according to the method previously described [40]. The methanol extract was subjected to liquid–liquid partition to obtain four fractions, hexane (7 g), ethyl acetate (3 g), butanol (~30 g), and water (~55 g).

The hexane extract was subjected to medium pressure liquid chromatography (MPLC) with a stepwise elution of hexane/acetone on SiO_2_-column to afford 8 fractions (Figure S2). The 4th fraction (335.9 mg) was separated by C_18_-MPLC (linear gradient of acetonitrile/aqueous formic acid) to obtain 2 major sub-fractions containing crude compounds **1** and **4**, respectively. Purified 0.6 mg of compound **1** was obtained by C_18_-HPLC separation using isocratic 99% acetonitrile. The 2nd sub-fraction (37.4 mg) was purified by C_18_-HPLC to afford compound **4** (30.0 mg). The 5th fraction (638.8 mg) was separated by C_18_-MPLC with a linear gradient of acetonitrile/aqueous formic acid to obtain a sub-fraction (136.3 mg) containing crude compound **2**. Compound **2** was further purified by SiO_2_-MPLC via a stepwise gradient of hexane/chloroform solvent system and C_18_-HPLC via isocratic 99% acetonitrile solvent system to obtain 2.4 mg white amorphous powder. The low polar fraction of the EtOAc extract (~3 g) from *P. polystachyus* was obtained after separation by SiO_2_-MPLC via a stepwise gradient of hexane/ethyl acetate solvent system. Further C_18_-MPLC separation with a linear gradient of acetonitrile/aqueous formic acid of this fraction afforded crude compound **3**, which was purified by C_18_-HPLC using isocratic 99% acetonitrile to obtain 6.1 mg as a white amorphous powder.

8ß,13ß-kaur-15-en-17-al, **1**: amorphous white powder; [α]_589_^23^ –106.8 ° (c 0.1, MeOH); UV λmax (MeOH) (log e) 257 (8721); ^1^H-NMR (500 MHz, CDCl_3_): δ 0.73 (3H, s, H-20), δ 0.82 (3H, s, H-19), δ 0.86 (3H, s, H-18), δ 0.88 (1H, m, H-5), δ 1.10 (1H, m, H-11), δ 1.20 (1H, m, H-9), δ 1.25 (1H, m, H-14), δ 1.38 (1H, m, H-2), δ 1.40 (1H, m, H-6), δ 1.40 (1H, m, H-7), δ 1.44 (1H, m, H-12), δ 1.47 (1H, m, H-2), δ 1.58 (1H, m, H-11), δ 1.58 (1H, m, H-12), δ 1.61 (1H, m, H6), δ 1.71 (1H, m, H-7), δ 1.82 (1H, d, J = 8.0 Hz, 5.7 Hz, 2.3 Hz, H-14), δ 2.85 (1H, m, H-13), 6.85 (1H, s, H-15), δ 9.71 (1H, s, H-17); ^13^C-NMR (125 MHz, CDCl_3_): See Table 1; HR-ESI-MS (m/z): 287.2365 [M+H]^+^ (calcd. for C_20_H_31_O, 287.2369).

### 3.4. Computer Calculation of ^13^C NMR Chemical Shifts

The NMR chemical shifts were calculated by Spartan’20 [41] (Spartan’20, Wavefunction, Inc.: Irvine, CA, USA). 8ß,13ß-Kaur-15-en-17-ol forming 5*S*, 8*S*, 9*R*, 10*S*, 13*R* conformer was used for the calculation. It was subjected to a conformational analysis using Merck molecular force field. The obtained conformers were optimized by DFT at the ωB97XD/6-31* level to afford the optimized conformers. Energy and Boltzmann distributions were calculated on the optimized conformers by DFT at the ωB97XV/6-311+G(2df,2p) level. The most optimized conformer covered 98.5% of all population with over 10 kJ/mol of energy difference from other ones and used for the simulation of NMR chemical shifts by DFT at the ωB97XD/6-31G* level to afford theoretical NMR chemical shifts.

### 3.5. Computer Calculation of ECD Spectra

8ß,13ß-Kaur-15-en-17-ol forming 5*S*, 8*S*, 9*R*, 10*S*, 13*R* conformer and its enantiomer were used for calculation. The conformational analysis and optimization of conformers were performed by Spartan’20 by the same procedure as described in the calculation of NMR chemical shifts. The optimized conformers were evaluated for their energy and Boltzmann distribution by DFT at the ωB97XV/6-311+G(2df,2p) level. The most optimized conformers for the 5S-form and enantiomer covered 98.5 and 98.4% of all populations with over 10 kJ/mol of energy differences from other conformers, respectively. They were selected for simulation of ECD spectra by TD-DFT at the CAM-B3LYP/6-31G(d) level with solvation of the IEFPCM model (MeOH) using Gaussian 16 C0.1 [42] to afford theoretical ECD spectra for 5S-form and enantiomer, respectively.

### 3.6. In Vitro Anti-Malarial Activity

The anti-malarial assay was conducted according to the previously described procedures [43]. Prior to the assay, samples were prepared in dimethyl sulfoxide (DMSO). The solubility of the compounds was at least more than 10 mg/mL, and any precipitation was not observed during the assay. *Plasmodium falciparum* 3D7 were cultured under 5.0% CO_2_ and 5% O_2_ in 3% hematocrit-type A human red blood cells (Japanese Red Cross Society) at 37 °C in RPMI1640 containing 25 mM HEPES, 24 mM NaHCO_3_ and 0.03% L-glutamine (Thermo Fisher Scientific), supplemented with 0.4% glucose, 20 μg/mL hypoxathine, 24 μg/mL gentamicin, and 0.25% AlbuMax II (Sigma-Aldrich). To perform *P. falciparum* growth assay, 50 μL of 0.3%-parasitized red blood cells and 2% hematocrit were dispensed into 384-well plate. Following 72 h exposure to a test sample, plates were frozen at −70 °C overnight and then thawed at room temperature for at least 4 h. To evaluate lactate dehydrogenase (LDH) activity, 25 μL of freshly made reaction mix (300 mM sodium L-lactate, 300 μM 3-acetyl pyridine adenine dinucleotide, 374 μM Nitro Blue tetrazolium chloride, 270 μg/mL diaphorase (22.5 U/mL), 1.5% Tween 20, 209 mM Tris-HCl, pH 8.0) was added. Plates were shaken to ensure mixing and absorbance at 620 nm was monitored in a plate reader after 10 min of incubation at room temperature. Artemisinin was used as a positive control for this assay with an IC_50_ of 14 ± 0.4 nM against the 3D7 strain of *P. falciparum*. The infection rates and IC_50_ of the samples are summarized in Tables S1 and S2.

### 3.7. In silico Anti-Malarial Activity

#### 3.7.1. Molecular Docking Simulation

The crystal structure of *P. falciparum* lactate dehydrogenase (*Pf*LDH) in complex with 3,5-dihydroxy-2-naphthoic acid (PDB ID: 1U5A) [32] was retrieved from the Protein Data Bank database [44]. Using Biovia Discovery Studio Visualizer, all water and heteroatoms were removed [45]. The PDB2PQR web service accessed on 14 March 2022 (https://pdb2pqr.poissonboltzmann.org/pdb2pqr/) was used to perform additional calculations on the crystal structure. Reconstruction of missing atoms and assignment of atomic charges and radii using the SWANSON force field (AMBER ff99 charges with optimized radii) was performed [46]. Protonation at pH 7.40 was carried out using (PROPKA3) [47,48]. The MolProbity web service was accessed on 14 March 2022 (http://molprobity.biochem.duke.edu/) for correction of bad contacts, the addition of missing hydrogen atoms, flipping of HIS, GLU, and ASN residues [48,49]. 

The derivatives (ligands) were sketched using PerkinElmer ChemDraw 17.1 (Figure 1). Their geometries were optimized using PerkinElmer Chem3D 17.1 via Molecular Mechanics 2 force field (MM2) and finally saved in PDB format [50,51]. 

Both the ligands and enzyme were prepared for docking using AutoDockTool 1.5.6 (The Scripps Research Institute, La Jolla, CA, USA) [52]. The enzyme was given polar hydrogens and Kollman charges, and the ligands were given Gasteiger charges and saved in PDBQT format. Ligand’s flexibility (active rotatable bonds) was preserved [52]. The parameters were set as following: grid box size = 40 × 40 × 40, grid spacing = 0.375 Å, and coordinates, x = 23.67, y = 18.31, z = 4.89 (centered on the binding pocket), which were saved in grid parameter files (GPFs). For docking, the enzyme was set as rigid and ligand as flexible, the number of genetic algorithm runs was set to 100, population size 150, the maximum number of evals was 2,500,000 (medium), the maximum number of generations was 27,000, the Lamarckian genetic algorithm was chosen to perform this process, and the remaining parameters were kept as default and saved in the docking parameter files (DPFs). AutoDock 4.2 was utilized to simulate the docking process [53]. The molecular interactions in the binding site were visualized and analyzed using BIOVIA Discovery Studio Visualizer [45].

#### 3.7.2. Molecular Dynamic Simulation

The best-docked conformation pose of the selected compounds (**1** and **2**) in the *Pf*LDH binding site was chosen as the starting structure to run the molecular dynamic (MD) simulations (100 ns). Using AMBER 18 (University of California San Francisco, CA, USA) [54], the MD simulations were performed based on the methods previously described [48,50]. Briefly, the general AMBER force field (GAFF) and the AMBER ff14SB force field were applied, respectively, on the ligands and *Pf*LDH. Using the ANTECHAMBER tools, the ligands were added with AM1-BCC model charges [55]. The enzyme was subjected to AMBER ff14SB force field. A TIP3P water model dipped in an octahedral box was employed to solve the systems, with a distance of 10 Å between the enzyme edge and the box. The chlorine atoms (counter ions) were added to neutralize the systems. Following solvation and neutralization, the values for each system were recorded: Apo-*Pf*LDH (ligand-free) had 43277 atoms, *Pf*LDH-**1** had 43315 atoms, *Pf*LDH-**2** had 43322 atoms, and *Pf*LDH-control had 43293 atoms.

Three minimization steps were conducted, consisting of 5000, 2000, and 1000 conjugate gradient cycles. Minimization of the collision contacts between the macromolecule and the solvent and relaxation of the system was achieved by using a constant volume of the periodic boundary conditions. The system was then gradually heated for 1 ns in three steps from 0–310 K on all backbone atoms using the Langevin dynamics thermostat (coupling time of 0.2 ps). The NVT ensemble was employed during the heating process. Then, the enzyme atoms and surrounding solvent were equilibrated in three steps of 2 ns each, and the SHAKE algorithm was used to restrict all hydrogen bonds [56].

The MD simulations were run until 100 ns. CPPTRAJ was used to conduct the trajectory analysis that examined the Root Mean Square Deviation and Fluctuation (RMSD and RMSF) values, radius of gyration (Rg) and hydrogen bond (H-bond). To illustrate their graphs, QtGrace 0.2.6 was employed. Using the MM-PBSA.py module in AMBER 18, free binding energy and its energetic components for each system were recorded for every 10 ps (total 1000 snapshots) from the last 10 ns of the trajectory. The data were calculated based on the “PBSA” model [57]. To give a closer approximation to the true molecular volume, albeit in an average sense, a salt concentration of 0.150 M was set without quasi-harmonic entropy approximation. All the equations used in this study were described in detail in our previous work [48,50].

## 4. Conclusions

A new natural product 8ß,13ß-kaur-15-en-17-al (**1**) along with two known kaurene diastereomers and one vitamin E derivative were isolated in this study. Compounds **1** and **2** exerted fair inhibition against the 3D7 strain of *P. falciparum*, with the lowest IC_50_ at 12 µM. Evidence of molecular interactions and strong binding free energies were observed between **1** and **2** with the putative target *Pf*LDH in the in silico molecular docking. The MD simulations unveiled that the dynamic stability, structural stability, and molecular interactions including H-bonds and van der Waals interactions were achieved in their simulated systems. This study delivers a preliminary result for estimating the mechanism of inhibition of **1** and **2**, warranting in vitro anti-*Pf*LDH activity and in vivo studies of the kaurene derivatives as anti-malarial agents in the future. Additionally, a comparative study with reference to anti-malarial drugs is worthy of further investigation. On the whole, it rationalizes the relevance of the kaurene derivatives as plasmodial growth inhibitors, which could serve as a potential scaffold for the anti-malarial drug design. 

## Figures and Tables

**Table 1:** ^13^C and ^1^H NMR spectral data of **1** and its diastereomer, *ent*-kaur-15-en-17-al (CDCl_3_ solution, *δ* values in ppm, *J* values in Hz).

^13^C	1		*ent*-Kaur-15-en-17-al [22]		Calculated NMR
	*δ*^13^C	*δ*^1^H (*J*)	*δ*^13^C	*δ*^1^H (*J*)	*δ*^13^C
**1**	38.8		40.4		38.5
**2**	18.8	1.38, m 1.47, m	18.5		19.2
**3**	42.2		42.0		40.6
**4**	33.4		33.3		33.0
**5**	56.0	0.88, m	55.9	0.80, dd (12,2)	55.7
**6**	20.2	1.40, m 1.61, m	18.7		21.2
**7**	36.3	1.40, m 1.71, m	38.2		36.3
**8**	50.0		51.0		49.4
**9**	54.20	1.20, m	46.8	1.06, bd (12)	53.6
**10**	37.7		39.8		38.1
**11**	19.1	1.10, m 1.58, m	18.4		19.7
**12**	24.9	1.44, m 1.58, m	25.3		24.5
**13**	36.4	2.85, m	38.0	3.01, bd (5)	36.4
**14**	54.17	1.25, m 1.82, ddd (8.0, 5.7, 2.3)	43.0	1.36, dd (10.5,5) 2.17, dd (10.5,1)	54.3
**15**	157.5	6.85, s	162.3	6.55, s	156.8
**16**	147.6		148.5		147.9
**17**	190.1	9.71, s	189.5	9.70, s	191.4
**18**	22.1	0.86, s	33.5	0.85, s	22.7
**19**	33.8	0.82, s	21.5	0.79, s	34.0
**20**	15.7	0.73, s	17.7	1.04, s	17.1

**Table 2:** The docking scores for compounds **1**, **2**, and the co-crystallized ligand (3,5-dihydroxy-2-naphthoic acid) in the active binding site of *Pf*LDH (1U5A.PDB) using AutoDock 4.2.

Compound	*ΔG_bind_ (kcal/mol)	Experimental IC_50_ (µM)
**1**	−8.03	12
**2**	−7.97	52
Control	−5.94	---

**Table 3:** The binding free energies (MM-PBSA) for complexes from MD simulation trajectories (90–100 ns). The molecular docking scores from AutoDock 4.2 for the complexes are compared in the table.

Complex with *Pf*LDH	ΔG_bind_ kcal/mol	VDW kcal/mol	EEL kcal/mol	G_polar_ kcal/mol	G_non-polar_ kcal/mol	AutoDock 4.2 kcal/mol
**1**	−30.87 ± 0.15	−30.33 ± 0.09	−7.06 ± 0.81	9.87 ± 0.14	−3.96 ± 0.01	−8.03
**2**	−24.70 ± 0.15	−35.85 ± 0.09	−7.37 ± 0.14	22.92 ± 0.11	−4.40 ± 0.07	−7.97
Control	−16.64 ± 0.12	−15.07 ± 0.14	−116.83 ± 0.51	117.49 ± 0.47	−2.23 ± 0.01	−5.94

ΔG_bind_*:* binding free energy, VDW: van der Waals, EEL: electrostatic, G_polar_: polar solvation energy, G_non-polar_: non-polar solvation energy.

## Data Availability

Data are contained within the article and Supplementary Material.

## Supplementary Materials

https://www.mdpi.com/article/10.3390/ph15070902/s1. Figure S1: isolation scheme of the leaves extract from *P. polystachyus*; Figure S2: HR-ESI-TOF-MS spectrum of **1**; Figure S3: HR-ESI-TOF-MS spectrum of **2**; Figure S4: HR-ESI-TOF-MS spectrum of **3**; Figure S5: HR-EI-TOF-MS spectrum of **4**; Figure S6: ^1^H NMR spectrum of **1**; Figure S7: ^13^C NMR spectrum of **1**; Figure S8: DEPT-135 spectrum of **1**;Figure S9: HSQC spectrum of **1**; Figure S10: DQF-COSY spectrum of **1**; Figure S11: HMBC spectrum of **1**; Figure S12: NOESY spectrum of **1**; Figure S13: conformation analysis and calculation of NMR chemical shifts of 8ß,13ß-kaur-15-en-17-al; Figure S14: conformation analysis and calculation of ECD spectra of enantiomer of 8ß,13ß-kaur-15-en-17-al; Figure S15: experimental and calculated ECD spectra of **1**; Figure S16: ^1^H NMR spectrum of **2**; Figure S17: ^13^C NMR spectrum of **2**; Figure S18: DEPT-90 and DEPT-135 experiments of **2**; Figure S19: HSQC spectrum of **2**; Figure S20: DQF-COSY spectrum of **2**; Figure S21: HMBC spectrum of **2**; Figure S22: ^1^H NMR spectrum of **3**; Figure S23: ^13^C NMR spectrum of **3**; Figure S24: DEPT-135 spectrum of **3**; Figure S25: HSQC spectrum of **3**; Figure S26: DQF-COSY spectrum of **3**; Figure S27: HMBC spectrum of **3**; Figure S28: NOESY spectrum of **3**; Figure S29: ^1^H NMR spectrum of **4**; Figure S30: ^13^C NMR spectrum of **4**; Figure S31: DEPT-135 spectrum of **4**; Figure S32: HSQC-TOCSY spectrum of **4**; Figure S33: DQF-COSY spectrum of **4**; Figure S34: HMBC spectrum of **4**; Figure S35: NOESY spectrum of **4**; Figure S36: superimposition of the crystal structure of 3,5-dihydroxy-2-naphthoic acid (C pink and O red) and the docked model (C green and O red) on *Plasmodium falciparum* lactate dehydrogenase crystal structure (PDB ID: 1U5A) with RMSD ~ 2.0 Å and their 2D interactions into the binding site; Figure S37: hydrogen bond profiles obtained from MD simulations (0–100 ns) for all systems: (a) *Pf*LDH-apo, (b) *Pf*LDH- control, (c) *Pf*LDH-**1**, and (d) *Pf*LDH-**2**; Figure S38: Specimen ID and sample report of *P. polystachyus*.
