# Supplementary material for: Naturally Occurring 8ß,13ß-kaur-15-en-17-al and Anti-Malarial Activity from Podocarpus polystachyus Leaves
Source: Pharmaceuticals (Basel). 2022 Jul 21;15(7):902. doi: 10.3390/ph15070902 (PMC9318793; doi:10.3390/ph15070902)
Supplement: Supplementary file 1 [file pharmaceuticals-15-00902-s001.zip › pharmaceuticals-1794525-supplementary.pdf]

Supporting information

Naturally occurring 8ß,13ß-kaur-15-en-17-al and anti-malarial activity from *Podocarpus polystachyus* leaves

Mira Syahfriena Amir Rawa[a,b,c], Mohammad G. Al-Thiabat[a], Toshihiko Nogawa[c,d], Yushi Futamura[e], Akiko Okano[e], and Habibah A. Wahab[a,b,c,*]

[a]*School of Pharmaceutical Sciences, Universiti Sains Malaysia, 11800 Minden, Penang, Malaysia*

[b]*Collaborative Laboratory of Herbal Standardization (CHEST), School of Pharmaceutical Sciences, Universiti Sains Malaysia, 11900 Bayan Lepas, Malaysia*

[c]*USM-RIKEN Interdisciplinary Collaboration for Advanced Science (URICAS), Universiti Sains Malaysia, 11800 Minden, Penang Malaysia*

[d]*Molecular Structure Characterization Unit, Technology Platform Division, RIKEN Center for Sustainable Resource Science, 2-1 Hirosawa, Saitama 351-0198, Japan*

[e]*Chemical Biology Research Group, RIKEN Center for Sustainable Resource Science, 2-1 Hirosawa, Wako, Saitama 351-0198, Japan*

Correspondence

Prof. Habibah A. Wahab
School of Pharmaceutical Sciences, Universiti Sains Malaysia, 11800 Minden, Penang, Malaysia
Tel: +604-6577888
Email: habibahw@usm.my
Orcid ID: 0000-0002-8353-8679

Contents:

Figure S1. Isolation scheme of the leaves extract from *Podocarpus polystachyus*. / page 4
Figure S2. HR-ESI-TOF-MS spectrum of **1**. / page 5
Figure S3. HR-ESI-TOF-MS spectrum of **2**. / page 6
Figure S4. HR-ESI-TOF-MS spectrum of **3**. / page 7
Figure S5. HR-EI-TOF-MS spectrum of **4**. / page 8
Figure S6. ^{1}H NMR spectrum of **1**. / page 9
Figure S7. ^{13}C NMR spectrum of **1**. / page 10
Figure S8. DEPT-135 spectrum of **1**. / page 11
Figure S9. HSQC spectrum of **1**. / page 12
Figure S10. DQF-COSY spectrum of **1**. / page 13
Figure S11. HMBC spectrum of **1**. / page 14
Figure S12. NOESY spectrum of **1**. / page 15
Figure S13. Conformation analysis and calculation of NMR chemical shifts of 8β,13β-kaur-15-en-17-al. / page 16
Figure S14. Conformation analysis and calculation of ECD spectra of enantiomer of 8β,13β-kaur-15-en-17-al. / page 17
Figure S15. Experimental and calculated ECD spectra of **1**. / page 18
Figure S16. ^{1}H NMR spectrum of **2**. / page 19
Figure S17. ^{13}C NMR spectrum of **2**. / page 20
Figure S18. DEPT-90 and DEPT-135 experiments of **2**. / page 21
Figure S19. HSQC spectrum of **2**. / page 22
Figure S20. DQF-COSY spectrum of **2**. / page 23
Figure S21. HMBC spectrum of **2**. / page 24
Figure S22. ^{1}H NMR spectrum of **3**. / page 25
Figure S23. ^{13}C NMR spectrum of **3**. / page 26
Figure S24. DEPT-135 spectrum of **3**. / page 27
Figure S25. HSQC spectrum of **3**. / page 28
Figure S26. DQF-COSY spectrum of **3**. / page 29
Figure S27. HMBC spectrum of **3**. / page 30
Figure S28. NOESY spectrum of **3**. / page 31
Figure S29. ^{1}H NMR spectrum of **4**. / page 32
Figure S30. ^{13}C NMR spectrum of **4**. / page 33

Figure S31. DEPT-135 spectrum of **4**. / page 34

Figure S32. HSQC-TOCSY spectrum of **4**. / page 35

Figure S33. DQF-COSY spectrum of **4**. / page 36

Figure S34. HMBC spectrum of **4**. / page 37

Figure S35. NOESY spectrum of **4**. / page 38

Figure S36. Superimposition of the crystal structure of 3,5-dihydroxy-2-naphthoic acid (C pink and O red) and the docked model (C green and O red) on Plasmodium falciparum lactate dehydrogenase crystal structure (PDB ID: 1U5A) with RMSD ~ 2.0 Å and their 2D interactions into the binding site. / page 39

Figure S37. Hydrogen bond profiles obtained from MD simulations (0–100 ns) for all systems: (a) *Pf*LDH-apo, (b) *Pf*LDH- control, (c) *Pf*LDH-**1**, and (d) *Pf*LDH-**2**. / page 40

Figure S38. Specimen ID and sample report of *P. polystachyus*. / page 41

Table S1. Infection rates of compounds **1** and **2** in comparison to artemisinin as the reference drug. / page 42

Table S2. IC_{50} of compounds **1** and **2** in comparison to artemisinin as the reference drug. / page 42

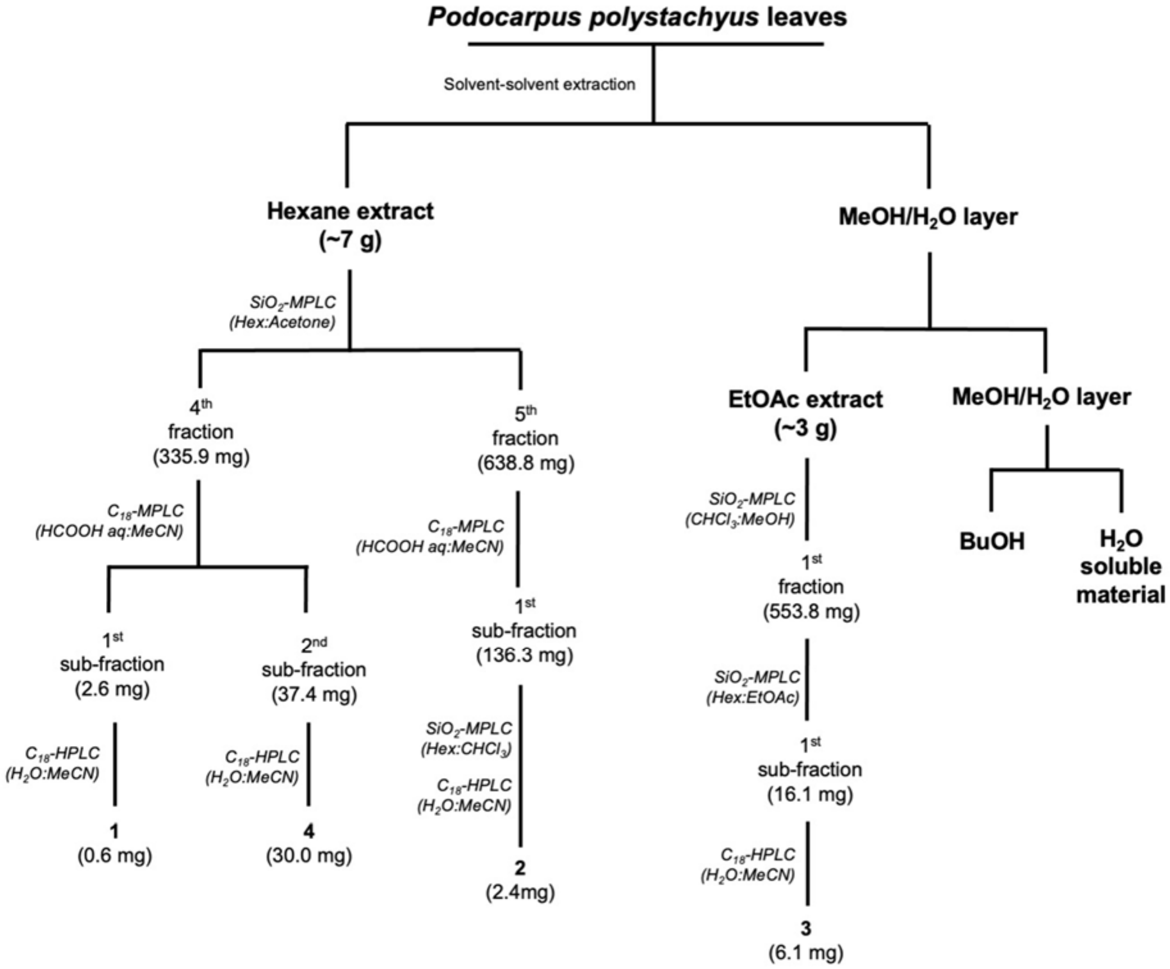

Figure S1. Isolation scheme of the leaf extract from *Podocarpus polystachyus*.

Compound **1**

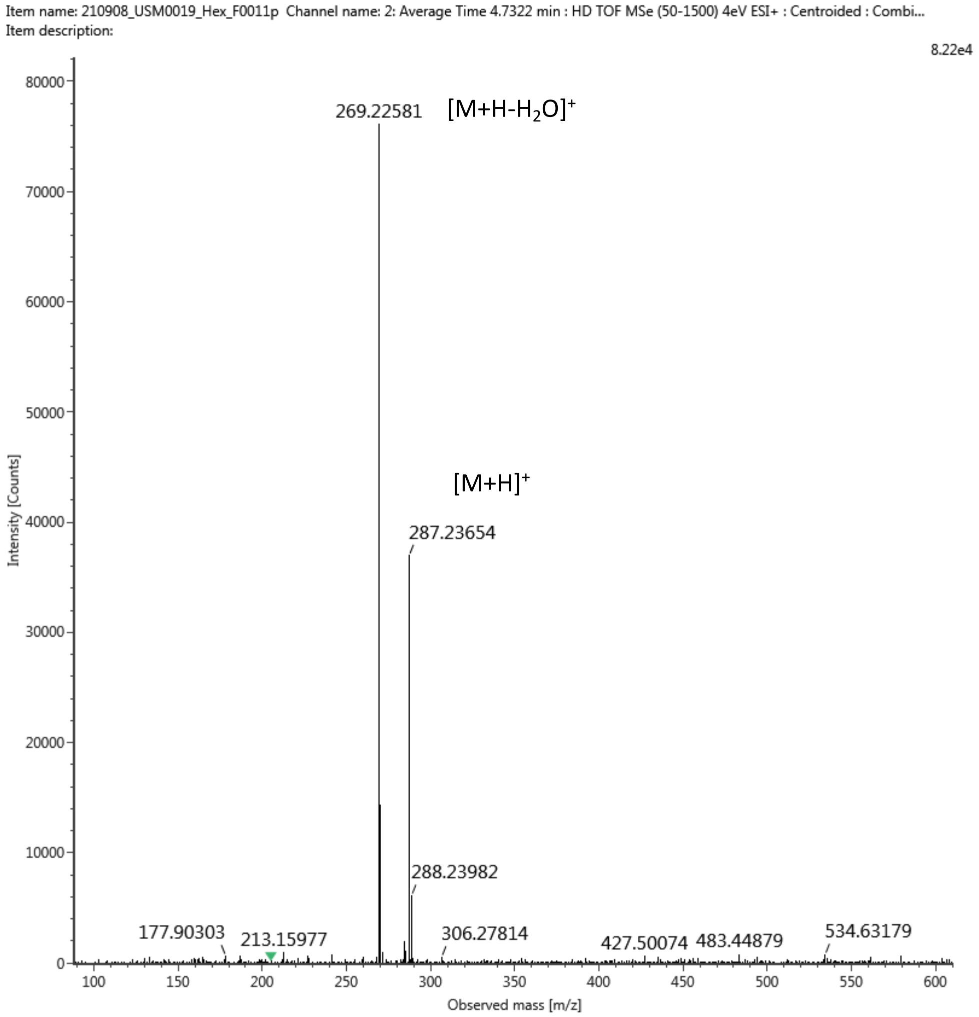

m/z: 287.23654 [M+H]$^+$

Composition	i-FIT Confidence (%)	m/z RMS (PPM)	Intensity RMS (%)	Predicted m/z	m/z error (PPM)	m/z error (mDa)	DBE
C20H30O	100.000000	1.468589	12.995479	287.236942	-1.404578	-0.402031	6.000000

m/z: 269.22581 [M+H-H_2O]$^+$

Composition	i-FIT Confidence (%)	m/z RMS (PPM)	Intensity RMS (%)	Predicted m/z	m/z error (PPM)	m/z error (mDa)	DBE
C20H28	100.000000	2.721461	7.787655	269.226377	-2.115235	-0.567345	7.000000

Figure S2. HR-ESI-TOF-MS spectrum of **1**.

Compound **2**

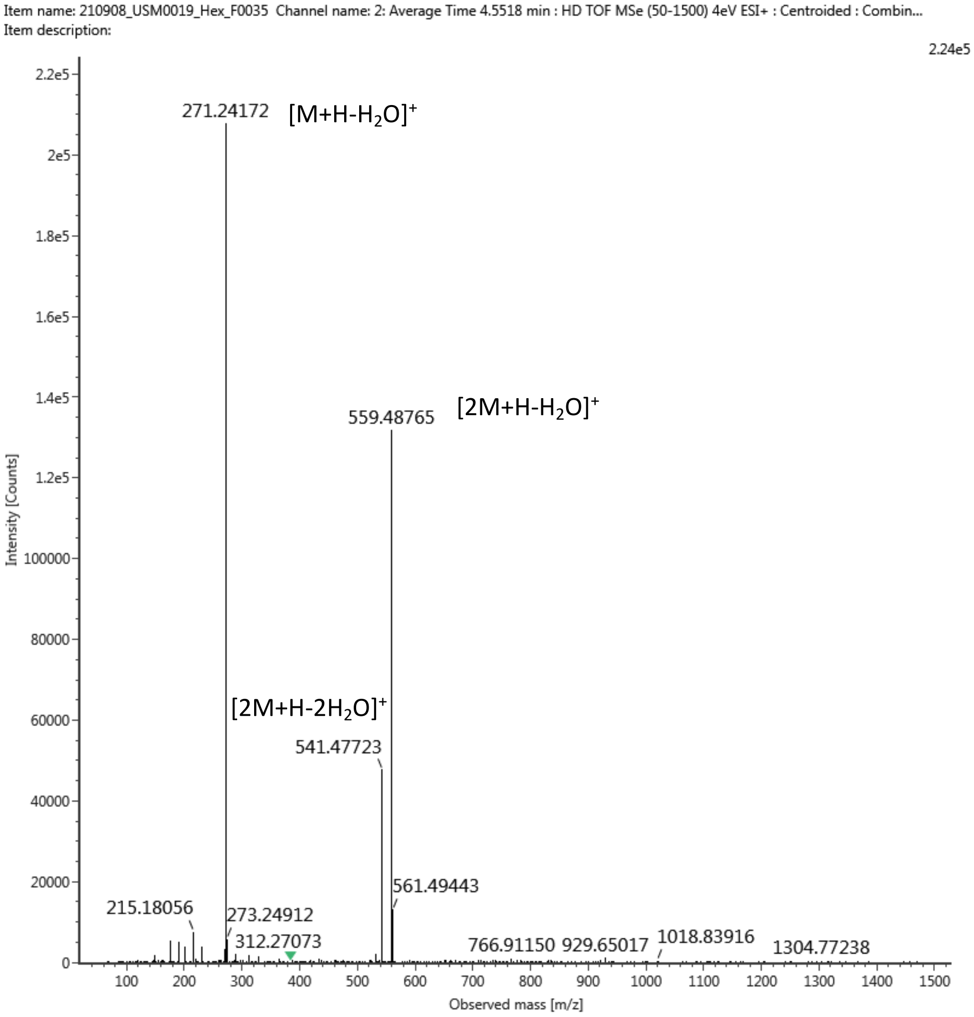

m/z: 271.24172 $[M+H-H_2O]^+$

Composition	i-FIT Confidence (%)	m/z RMS (PPM)	Intensity RMS (%)	Predicted m/z	m/z error (PPM)	m/z error (mDa)	DBE
C20H30	100.000000	1.490177	3.043532	271.242027	-1.137564	-0.307409	6.000000

m/z: 559.48765 $[2M+H-H_2O]^+$

Composition	i-FIT Confidence (%)	m/z RMS (PPM)	Intensity RMS (%)	Predicted m/z	m/z error (PPM)	m/z error (mDa)	DBE
C40H62O	100.000000	0.581055	1.098484	559.487343	0.549613	0.306948	10.000000

m/z: 541.47723 $[2M+H-2H_2O]^+$

Composition	i-FIT Confidence (%)	m/z RMS (PPM)	Intensity RMS (%)	Predicted m/z	m/z error (PPM)	m/z error (mDa)	DBE
C40H60	100.000000	1.078207	9.476290	541.476778	0.835632	0.451634	11.000000

Figure S3. HR-ESI-TOF-MS spectrum of **2**.

Compound **3**

Data:20210909_003-Profile
Comment:USM0019-EtOAc-F0009_0.1mg/mL+PFTBA,sless1uL(70-325i250,14min)
Description:
Ionization Mode:EI+(eiFi)
History:Centroid[Peak Detect[Centroid,30,Area];Smooth[3]];Average(MS[1] 11.10..11.24)

Acquired:2021/09/09 16:02:16
Operator:AccuTOF
m/z Calibration File:PFTBA800_EI_pos
Created:2021/09/09 16:36:17
Created by:AccuTOF

Charge number:1
Tolerance:1.00[ppm], 5.00 .. [mDa]
Unsaturation Number:-8.0 .. 80.0 (Fraction:...
Element:^{12}C:3 .. 20, ^{1}H:2 .. 32

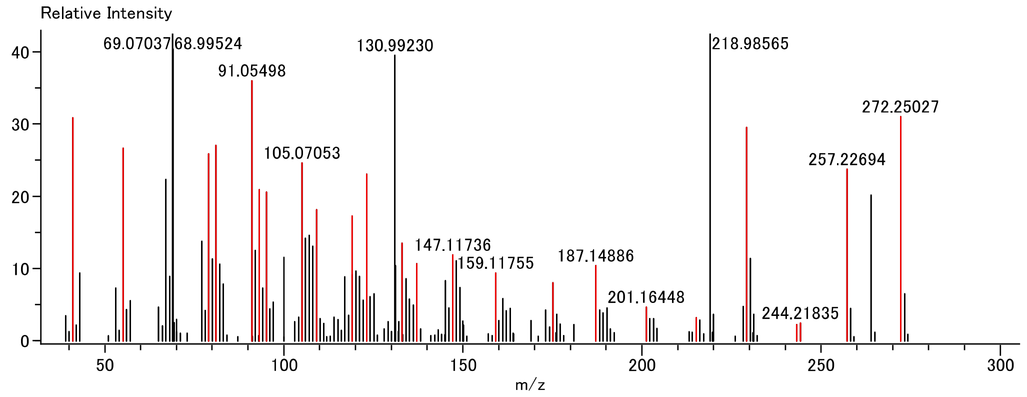

Mass	Intensity	Calc. Mass	Mass Difference [mDa]	Mass Difference [ppm]	Possible Formula	Unsaturation Number
41.03917	21306.96	41.03913	0.05	1.18	$^{12}C_3{}^1H_5$	1.5
55.05484	18356.58	55.05478	0.06	1.14	$^{12}C_4{}^1H_7$	1.5
79.05493	17847.33	79.05478	0.15	1.91	$^{12}C_6{}^1H_7$	3.5
81.07039	18665.05	81.07043	-0.03	-0.41	$^{12}C_6{}^1H_9$	2.5
91.05498	24823.68	91.05478	0.20	2.23	$^{12}C_7{}^1H_7$	4.5
93.07038	14401.98	93.07043	-0.04	-0.47	$^{12}C_7{}^1H_9$	3.5
95.08604	14167.45	95.08608	-0.04	-0.40	$^{12}C_7{}^1H_{11}$	2.5
105.07053	16966.44	105.07043	0.11	1.01	$^{12}C_8{}^1H_9$	4.5
109.10151	12509.90	109.10173	-0.21	-1.93	$^{12}C_8{}^1H_{13}$	2.5
119.08613	11896.73	119.08608	0.06	0.49	$^{12}C_9{}^1H_{11}$	4.5
123.11731	15913.46	123.11738	-0.06	-0.49	$^{12}C_9{}^1H_{15}$	2.5
133.10180	9297.61	133.10173	0.07	0.55	$^{12}C_{10}{}^1H_{13}$	4.5
137.13291	7333.20	137.13303	-0.12	-0.86	$^{12}C_{10}{}^1H_{17}$	2.5
147.11736	8160.18	147.11738	-0.02	-0.11	$^{12}C_{11}{}^1H_{15}$	4.5
159.11755	6419.89	159.11738	0.17	1.07	$^{12}C_{12}{}^1H_{15}$	5.5
175.14874	5522.66	175.14868	0.06	0.35	$^{12}C_{13}{}^1H_{19}$	4.5
187.14886	7151.34	187.14868	0.18	0.97	$^{12}C_{14}{}^1H_{19}$	5.5
201.16448	3169.76	201.16433	0.16	0.78	$^{12}C_{15}{}^1H_{21}$	5.5
215.18013	2149.29	215.17998	0.16	0.74	$^{12}C_{16}{}^1H_{23}$	5.5
229.19552	20388.62	229.19563	-0.10	-0.45	$^{12}C_{17}{}^1H_{25}$	5.5
243.21121	1500.19	243.21128	-0.07	-0.28	$^{12}C_{18}{}^1H_{27}$	5.5
244.21835	1645.92	244.21910	-0.75	-3.08	$^{12}C_{18}{}^1H_{28}$	5.0
257.22694	16372.79	257.22693	0.01	0.05	$^{12}C_{19}{}^1H_{29}$	5.5
272.25027	21381.51	272.25040	-0.13	-0.46	$^{12}C_{20}{}^1H_{32}$	5.0

Figure S4. HR-ESI-TOF-MS spectrum of **3**.

Compound **4**

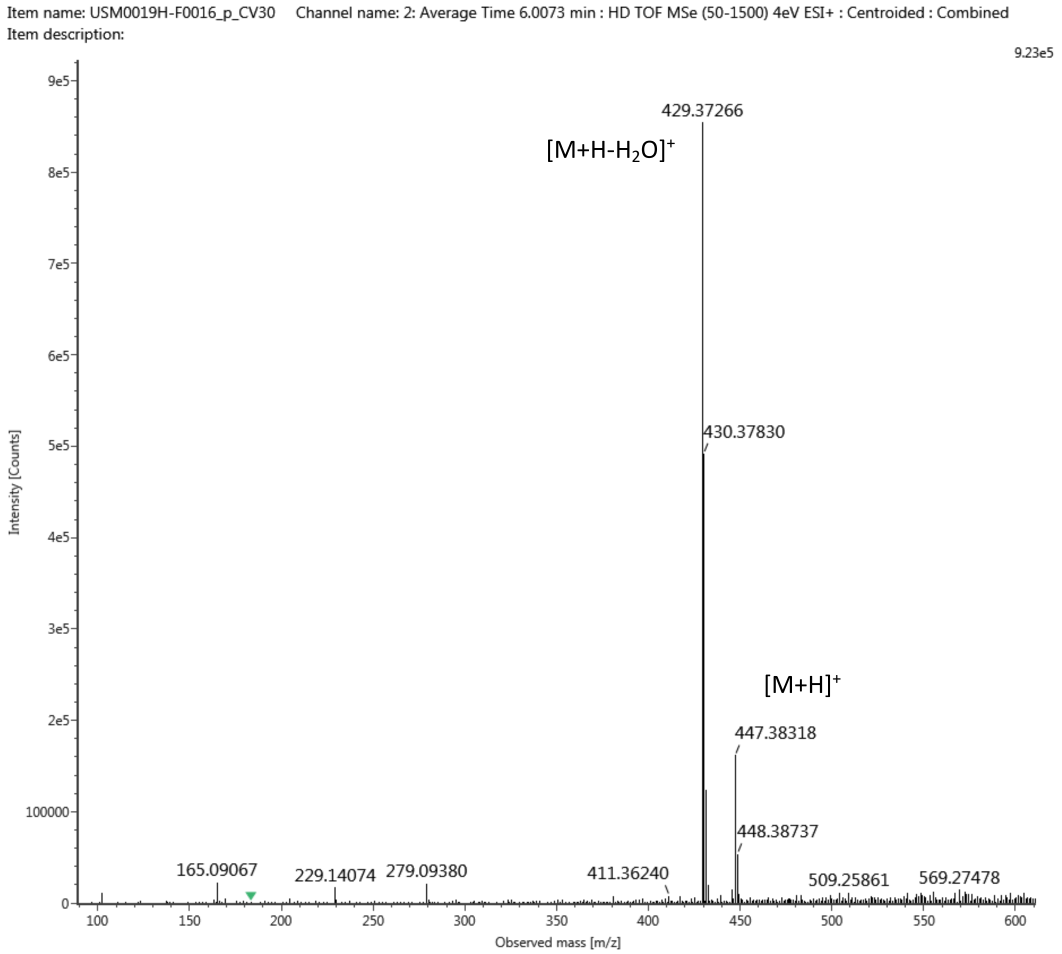

m/z: 447.38318 $[M+H]^+$

Composition	i-FIT Confidence (%)	m/z RMS (PPM)	Intensity RMS (%)	Predicted m/z	m/z error (PPM)	m/z error (mDa)	DBE
C29H50O3	100.000000	0.891141	3.402658	447.383272	-0.205911	-0.091914	5.000000

m/z: 429.37266 $[M+H-H_2O]^+$

Composition	i-FIT Confidence (%)	m/z RMS (PPM)	Intensity RMS (%)	Predicted m/z	m/z error (PPM)	m/z error (mDa)	DBE
C29H48O2	100.000000	2.798494	39.760358	429.372707	-0.110251	-0.047228	6.000000

Figure S5. HR-EI-TOF-MS spectrum of **4**.

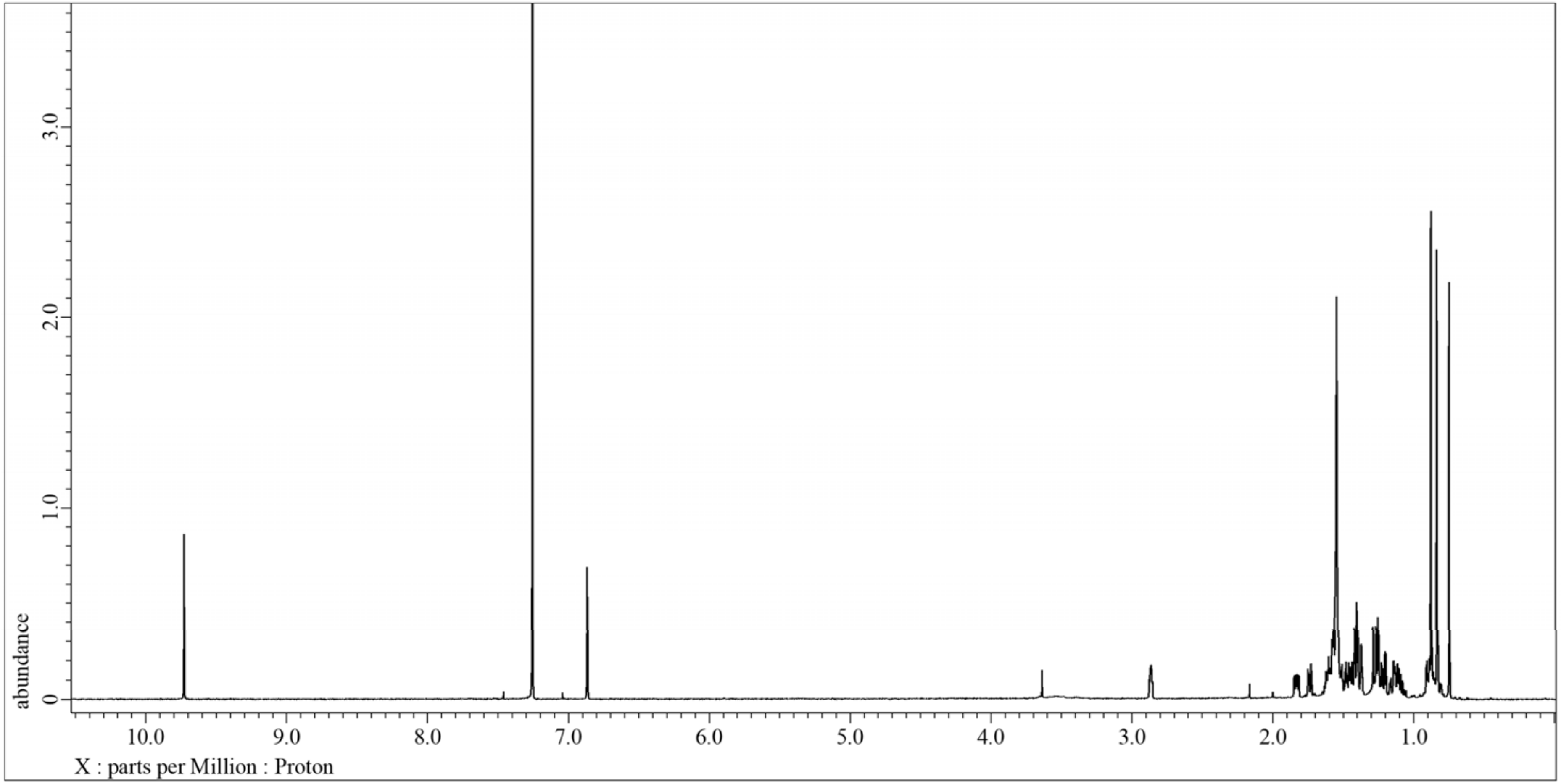

Figure S6. ^{1}H NMR spectrum of **1**.

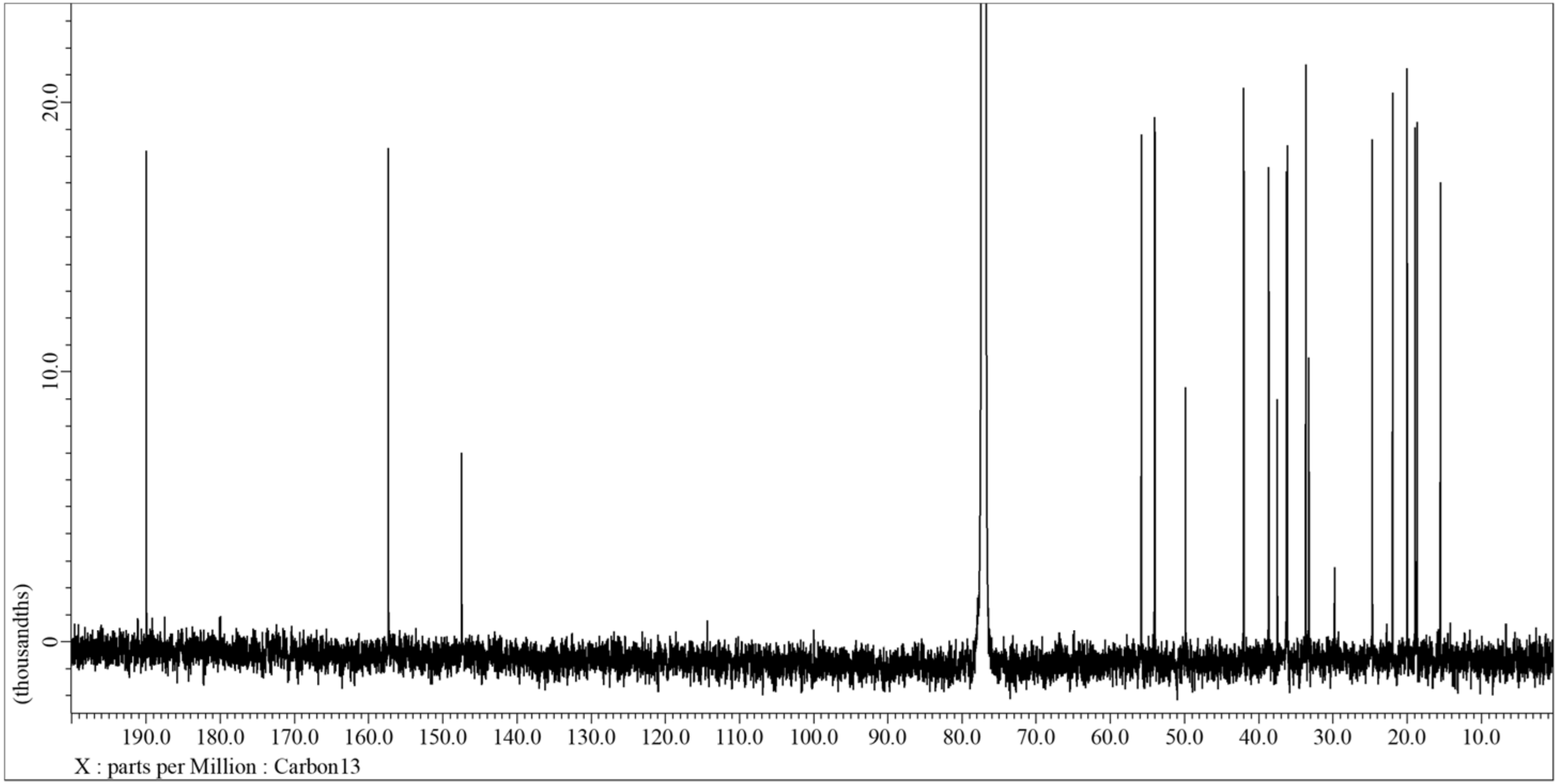

Figure S7. ^{13}C NMR spectrum of **1**.

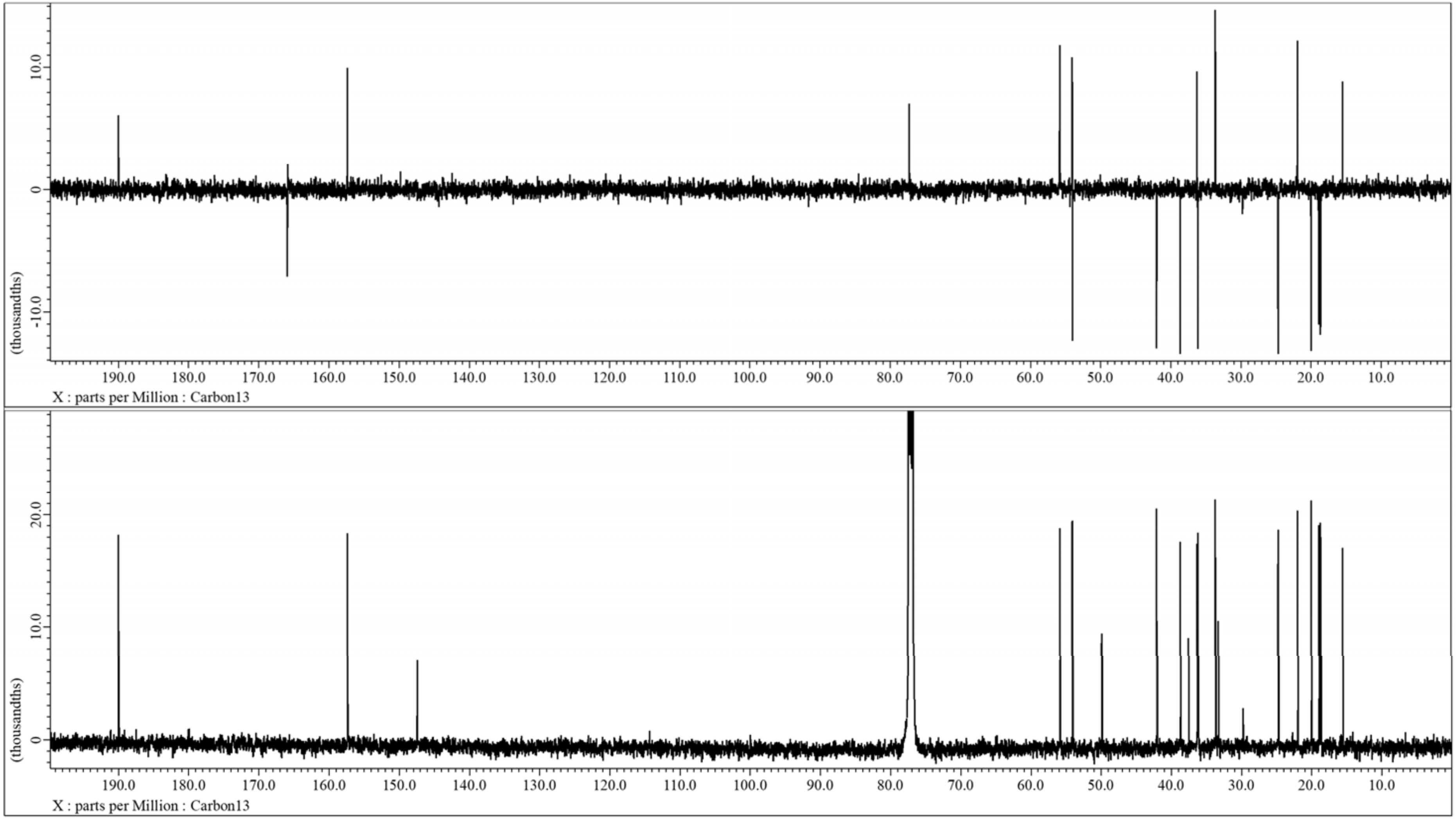

Figure S8. DEPT-135 spectrum of **1**.

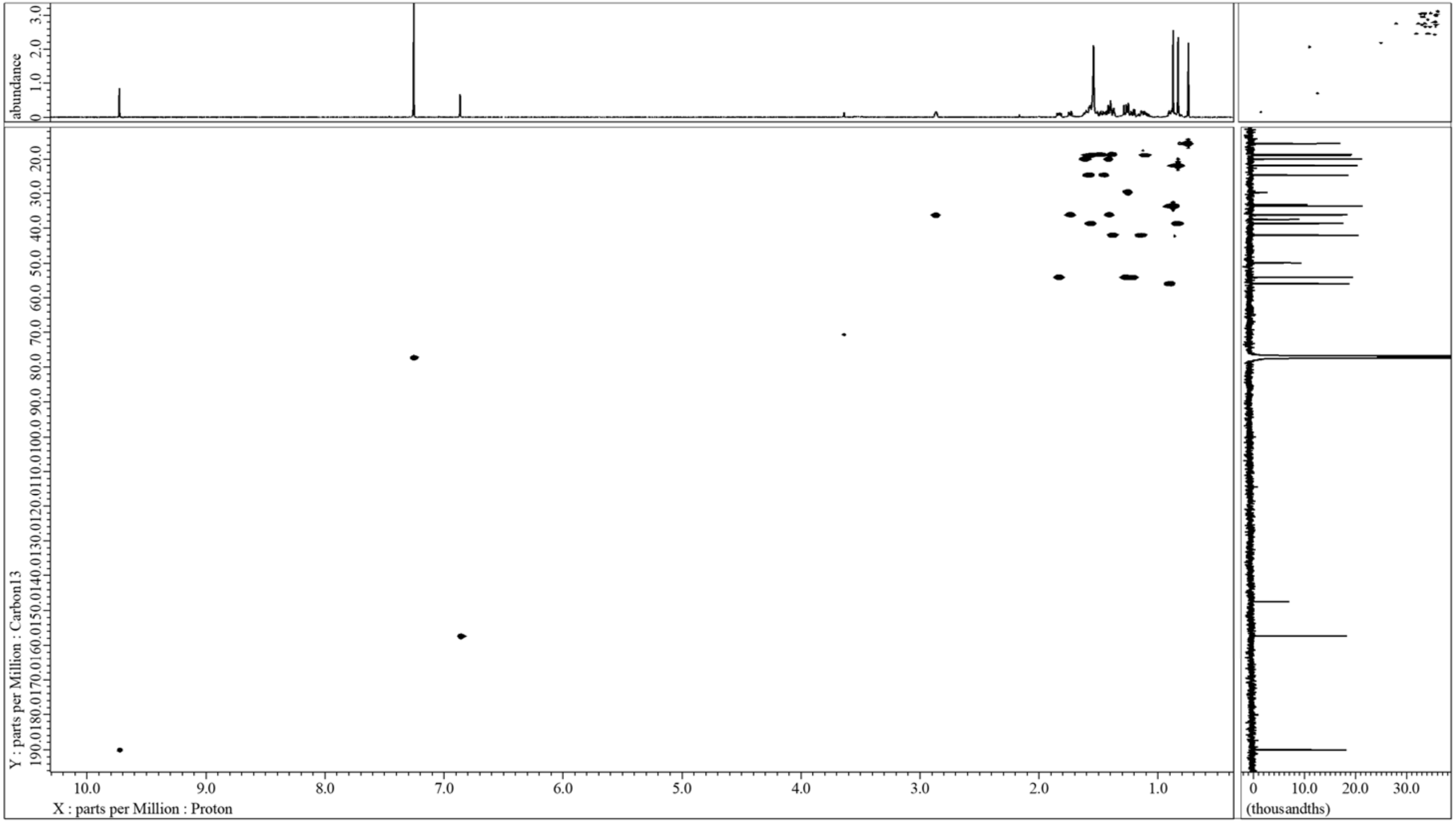

Figure S9. HSQC spectrum of **1**.

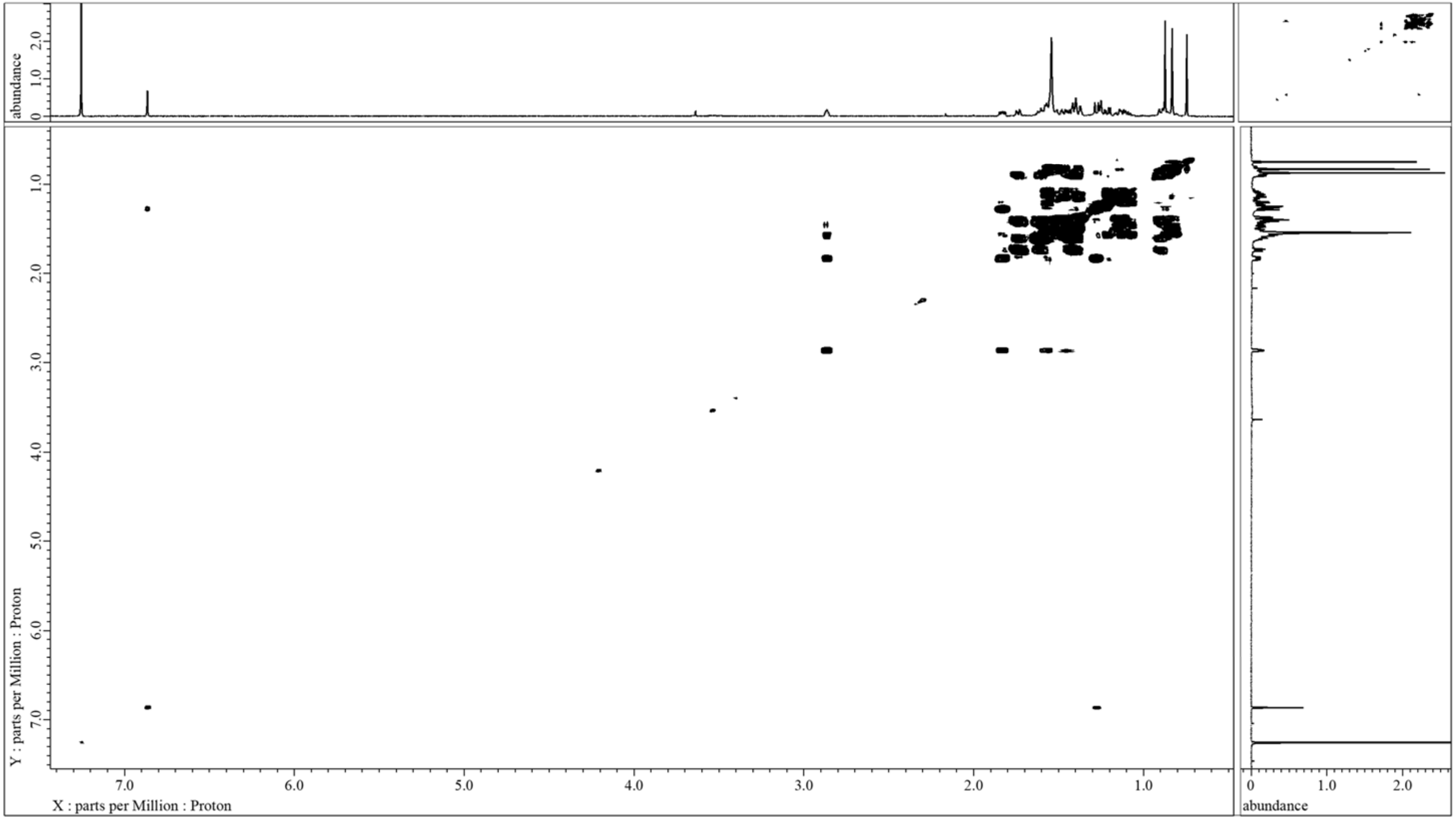

Figure S10. DQF-COSY spectrum of **1**.

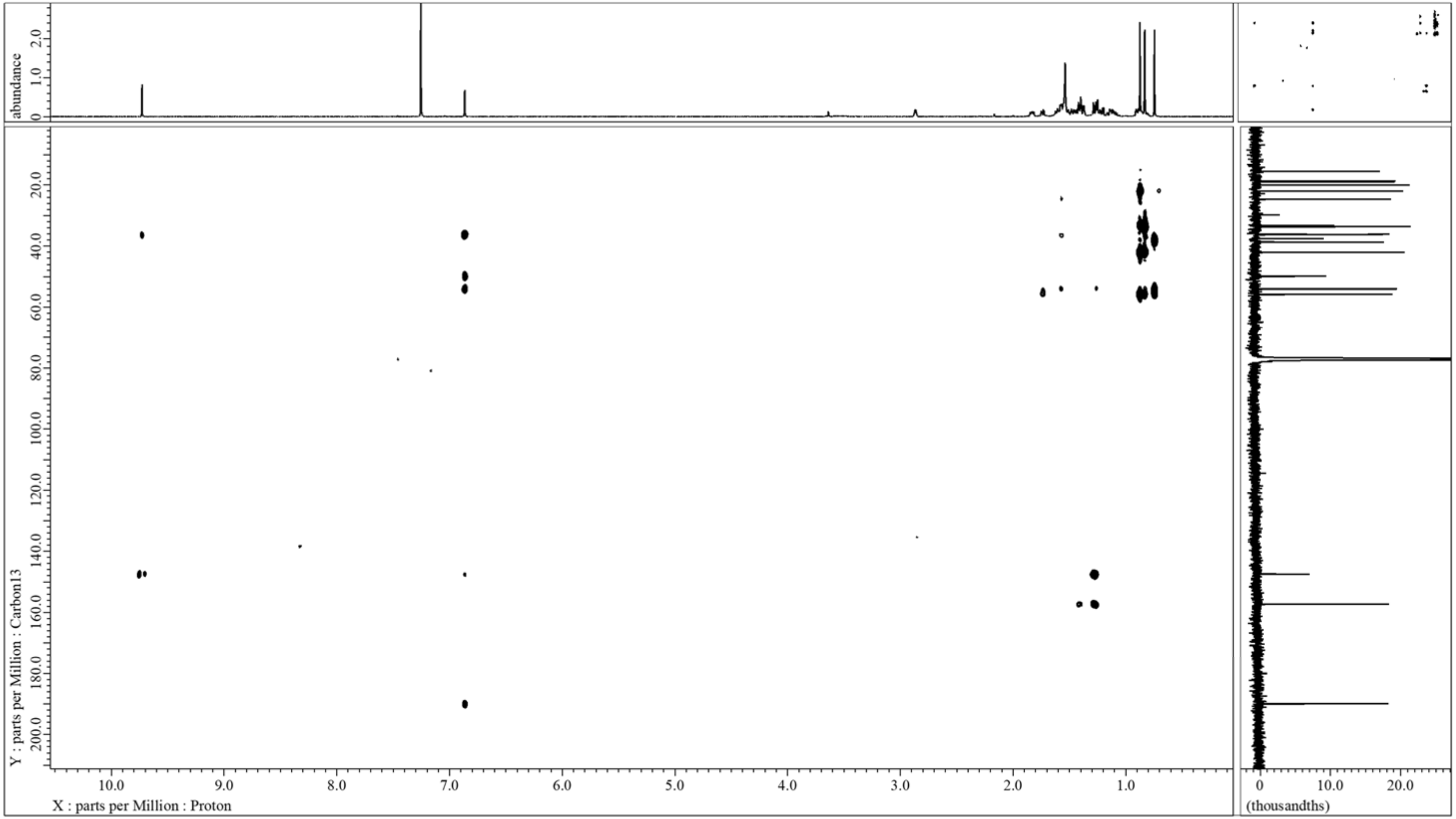

Figure S11. HMBC spectrum of **1**.

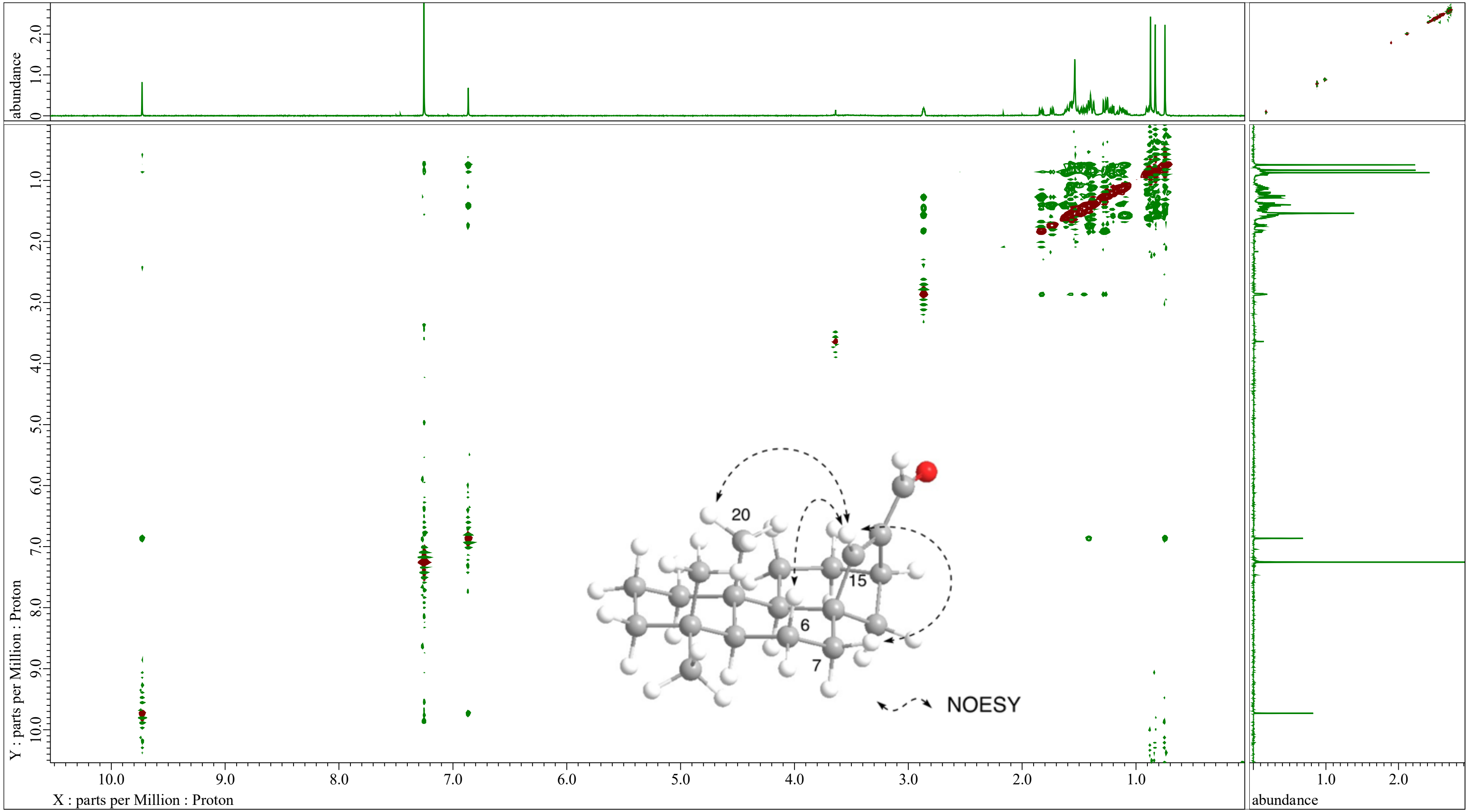

Figure S12. NOESY spectrum of **1**.

Starting structure				Optimized structure			
H	1.372017	-3.184947	-1.265359	H	1.390576	-3.135287	-1.247887
C	0.281771	-3.098313	-1.302125	C	0.295343	-3.074964	-1.266728
C	0.155735	-0.81795	-0.097608	C	0.174174	-0.78784	-0.094648
C	0.222443	-0.976649	-2.770696	C	0.217692	-0.977299	-2.756385
C	-0.234505	-0.189151	-1.490133	C	-0.219436	-0.186269	-1.485251
C	-0.155737	-2.471308	-2.618847	C	-0.181133	-2.45647	-2.577679
C	-0.245482	-2.318605	-0.099631	C	-0.21972	-2.281923	-0.065841
H	-0.101713	-4.124839	-1.253201	H	-0.061565	-4.10916	-1.196942
H	-1.246863	-2.577635	-2.6946	H	-1.278761	-2.523228	-2.611973
H	0.26266	-3.05479	-3.449052	H	0.190957	-3.036934	-3.43215
H	-1.340217	-2.405264	-0.090327	H	-1.317391	-2.356018	-0.045938
H	0.110325	-2.810446	0.813937	H	0.141829	-2.743046	0.860749
C	0.078433	1.322462	-1.524055	C	0.100088	1.31443	-1.536463
C	-0.708749	2.076734	-0.452013	C	-0.715444	2.070874	-0.490685
C	-0.543803	1.502135	0.956293	C	-0.56943	1.526399	0.930553
C	-0.69623	-0.057284	1.002438	C	-0.691173	-0.035611	0.973731
H	-0.199067	1.754207	-2.490646	H	-0.130435	1.722651	-2.525461
H	1.149045	1.51324	-1.405313	H	1.170497	1.493925	-1.381245
H	-1.771534	2.046818	-0.728899	H	-1.776831	2.002412	-0.768614
H	-0.419857	3.135438	-0.475945	H	-0.463052	3.13902	-0.495443
C	-0.549326	-0.614045	2.453017	C	-0.516341	-0.569134	2.415581
C	-1.19362	0.275936	3.538377	C	-1.220823	0.266416	3.500623
C	-1.011256	1.779172	3.276179	C	-1.054646	1.777321	3.252916
C	-1.607394	2.103054	1.90587	C	-1.651303	2.09482	1.877574
H	0.505694	-0.733161	2.716574	H	0.547904	-0.607488	2.665513
H	-0.990224	-1.615597	2.517339	H	-0.879847	-1.59987	2.468628
H	-0.774505	0.008572	4.51603	H	-0.82195	-0.006954	4.483629
H	-2.26684	0.051487	3.585176	H	-2.293793	0.038445	3.515129
H	-1.463037	2.391848	4.061496	H	-1.480811	2.35342	4.076933
H	-2.605041	1.671781	1.762252	H	-2.63428	1.639321	1.710822
H	-1.701007	3.190667	1.770175	H	-1.741901	3.177478	1.726562
C	-0.550242	-0.451207	-4.011211	C	-0.562741	-0.443207	-3.972776
H	-0.238844	0.559861	-4.291653	H	-0.21409	0.54426	-4.291584
H	-1.631042	-0.434626	-3.83162	H	-1.635896	-0.371434	-3.760245
H	-0.374002	-1.091704	-4.88386	H	-0.436643	-1.119857	-4.82575
H	-1.335353	-0.253197	-1.526275	H	-1.320465	-0.257751	-1.503934
H	-1.749624	-0.252218	0.741336	H	-1.735197	-0.235304	0.685254
C	1.718442	-0.834374	-3.107112	C	1.713728	-0.858674	-3.09131
H	2.025824	0.214938	-3.154151	H	2.028504	0.189748	-3.135021
H	2.370125	-1.347555	-2.401911	H	2.358557	-1.371207	-2.374688
H	1.936034	-1.273615	-4.088769	H	1.908324	-1.301349	-4.075695
C	1.670106	-0.729378	0.210763	C	1.680648	-0.67687	0.210622
H	2.265403	-1.40764	-0.399266	H	2.264219	-1.378	-0.387847
H	2.082247	0.268025	0.057396	H	2.084662	0.318308	0.014337
H	1.896029	-1.020158	1.239826	H	1.887802	-0.915972	1.257414
C	0.696972	1.98901	1.684244	C	0.673227	1.975271	1.68207
H	1.655726	2.168223	1.219332	H	1.651949	2.113848	1.230183
C	0.421627	2.126964	2.99061	C	0.401092	2.11454	2.987888
C	1.374296	2.556587	4.020398	C	1.388305	2.432619	4.019286
H	2.392694	2.784875	3.665138	H	2.407227	2.669991	3.643102
O	1.061464	2.653623	5.20008	O	1.147789	2.441601	5.209086

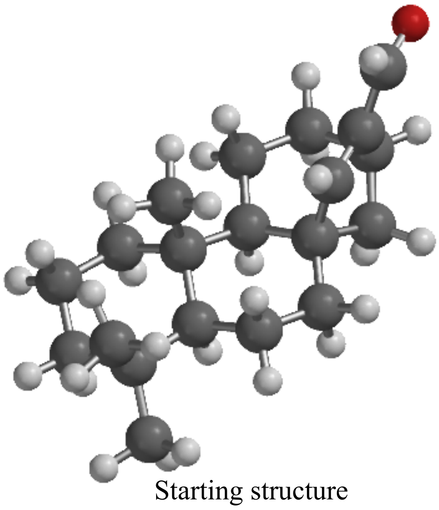

Starting structure
8β,13β-Kaur-15-en-17-al
(5*S*, 8*S*, 9*R*, 10*S*, 13*R*-form)

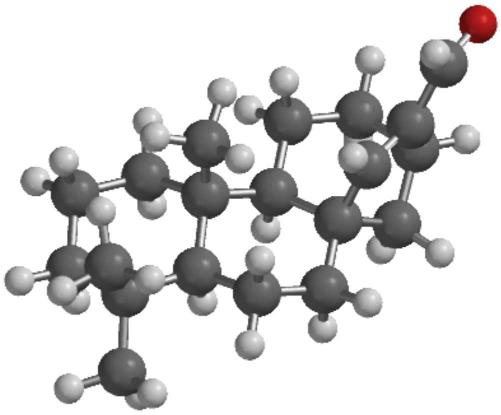

Optimized structure
8β,13β-Kaur-15-en-17-al
(5*S*, 8*S*, 9*R*, 10*S*, 13*R*-form)

Figure S13. Conformation analysis and calculation of NMR chemical shifts of 8β,13β-kaur-15-en-17-al.

Starting structure				Optimized structure			
H	-2.080519	1.232101	-1.320229	H	-2.020478	1.21494	-1.322955
C	-2.022778	0.148036	-1.15437	C	-1.978365	0.127525	-1.160398
C	-0.941188	0.363031	1.137383	C	-0.937473	0.34932	1.133461
C	-3.476718	0.285775	0.905948	C	-3.443419	0.305801	0.880529
C	-2.288475	-0.008343	1.855503	C	-2.279827	-0.00756	1.843088
C	-3.323148	-0.300162	-0.490978	C	-3.287212	-0.314031	-0.505116
C	-0.748853	-0.183013	-0.329466	C	-0.724377	-0.197291	-0.317117
H	-1.965101	-0.313056	-2.14794	H	-1.889221	-0.333728	-2.150971
H	-4.413484	-0.079804	1.345912	H	-4.388298	-0.01491	1.338613
H	-3.595903	1.373255	0.802116	H	-3.509093	1.397861	0.764881
H	-4.16705	0.030067	-1.109029	H	-4.129286	-0.01373	-1.13965
H	-3.388624	-1.391917	-0.455241	H	-3.336498	-1.408105	-0.441615
C	0.484803	0.596805	-0.949573	C	0.486322	0.598159	-0.916388
C	1.785959	0.520358	-0.078768	C	1.79157	0.552869	-0.048987
C	1.536929	0.841315	1.396245	C	1.509201	0.857929	1.421893
C	0.324078	0.123624	1.989345	C	0.309889	0.107184	1.994261
H	2.422299	0.604314	2.000263	H	2.413099	0.658035	2.011523
H	1.376841	1.922759	1.505456	H	1.313696	1.936089	1.510511
H	0.53791	-0.943607	2.098896	H	0.534804	-0.963437	2.067022
H	0.186622	0.509021	3.004373	H	0.139529	0.446713	3.020676
C	2.806807	1.517379	-0.676968	C	2.786333	1.563227	-0.665159
C	3.244844	0.782463	-1.944269	C	3.233046	0.822442	-1.930085
C	2.10527	0.871178	-2.971509	C	2.081245	0.896448	-2.94935
C	0.771398	0.290677	-2.452867	C	0.789067	0.265492	-2.397008
H	3.661961	1.672525	-0.002638	H	3.627884	1.716958	0.021188
H	2.374528	2.503415	-0.883828	H	2.32284	2.535524	-0.867668
H	4.183215	1.186742	-2.334419	H	4.162162	1.192771	-2.368725
H	1.949756	1.924099	-3.238981	H	1.910699	1.951888	-3.195025
H	2.386973	0.351368	-3.895299	H	2.360408	0.39185	-3.880763
H	0.797749	-0.788578	-2.629594	H	0.870378	-0.818341	-2.520232
H	-0.042584	0.677717	-3.076781	H	-0.054529	0.579931	-3.019354
C	-2.359283	-1.458618	2.368457	C	-2.366477	-1.460444	2.339191
H	-2.432202	-2.195621	1.570419	H	-2.432077	-2.189446	1.529081
H	-3.250816	-1.603848	2.991359	H	-3.261486	-1.585956	2.960398
H	-1.494229	-1.712292	2.989007	H	-1.499646	-1.722102	2.956013
C	-0.526902	-1.713879	-0.38815	C	-0.486359	-1.718897	-0.365751
H	-1.430478	-2.280164	-0.165831	H	-1.402467	-2.275565	-0.163949
H	0.237074	-2.064236	0.305957	H	0.25581	-2.060327	0.358737
H	-0.236163	-2.04737	-1.387559	H	-0.146368	-2.031556	-1.35686
C	2.569974	-0.75924	-0.312081	C	2.554279	-0.7345	-0.315319
H	2.468566	-1.661537	0.27362	H	2.423131	-1.657024	0.244116
C	3.37626	-0.600415	-1.373268	C	3.363903	-0.583784	-1.37376
C	-2.485041	0.899281	3.100328	C	-2.453816	0.902238	3.074351
H	-1.775153	0.661549	3.898712	H	-1.779747	0.629552	3.892486
H	-2.365639	1.958366	2.845885	H	-2.27509	1.954509	2.823715
H	-3.490156	0.775913	3.521442	H	-3.476741	0.820417	3.459651
H	-1.003493	1.458424	1.021172	H	-1.000396	1.4428	0.999121
H	0.192493	1.659941	-0.939106	H	0.175464	1.654372	-0.885118
C	4.273008	-1.624081	-1.921509	C	4.170374	-1.647808	-1.971284
H	4.270342	-2.593654	-1.396742	H	4.16635	-2.612679	-1.418953
O	4.978321	-1.418064	-2.900803	O	4.807261	-1.521621	-2.996949

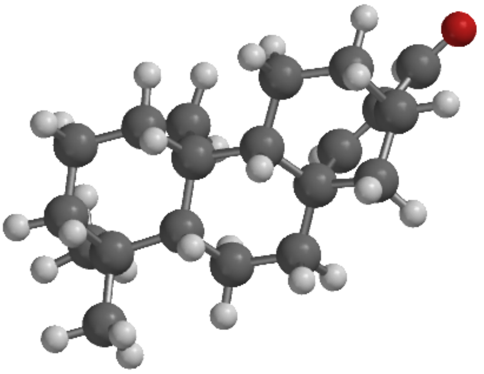

Starting structure
enantiomer of 8β,13β-Kaur-15-en-17-al
(5*R*, 8*R*, 9*S*, 10*R*, 13*S*-form)

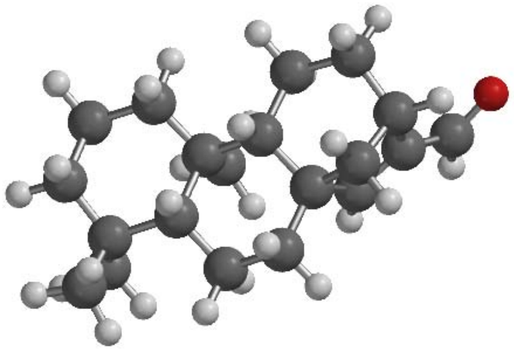

Optimized structure
enantiomer of 8β,13β-Kaur-15-en-17-al
(5*R*, 8*R*, 9*S*, 10*R*, 13*S*-form)

Figure S14. Conformation analysis and calculation of ECD spectra of enantiomer of 8β,13β-kaur-15-en-17-al. 8β,13β-Kaur-15-en-17-al (5*S*, 8*S*, 9*R*, 10*S*, 13*R*-form) was the same as NMR calculation. The stating and optimized structures of the enantiomer, 5*R*, 8*R*, 9*S*, 10*R*, 13*S*-form, was following the table.

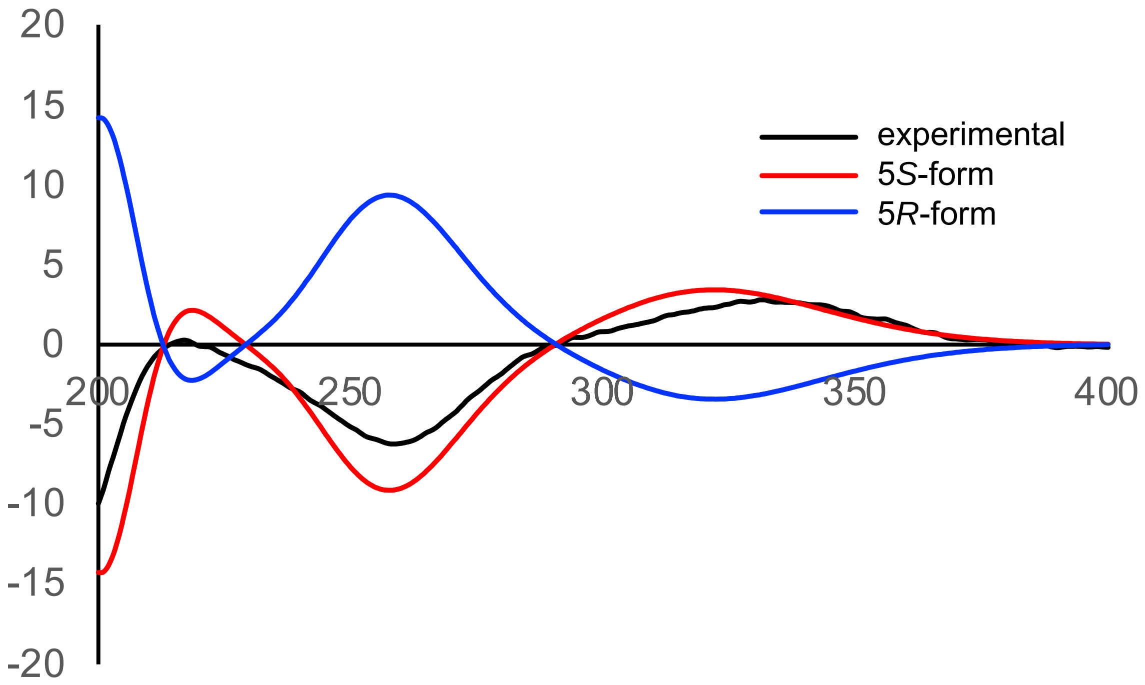

Figure S15. Experimental and calculated ECD spectra of **1**.

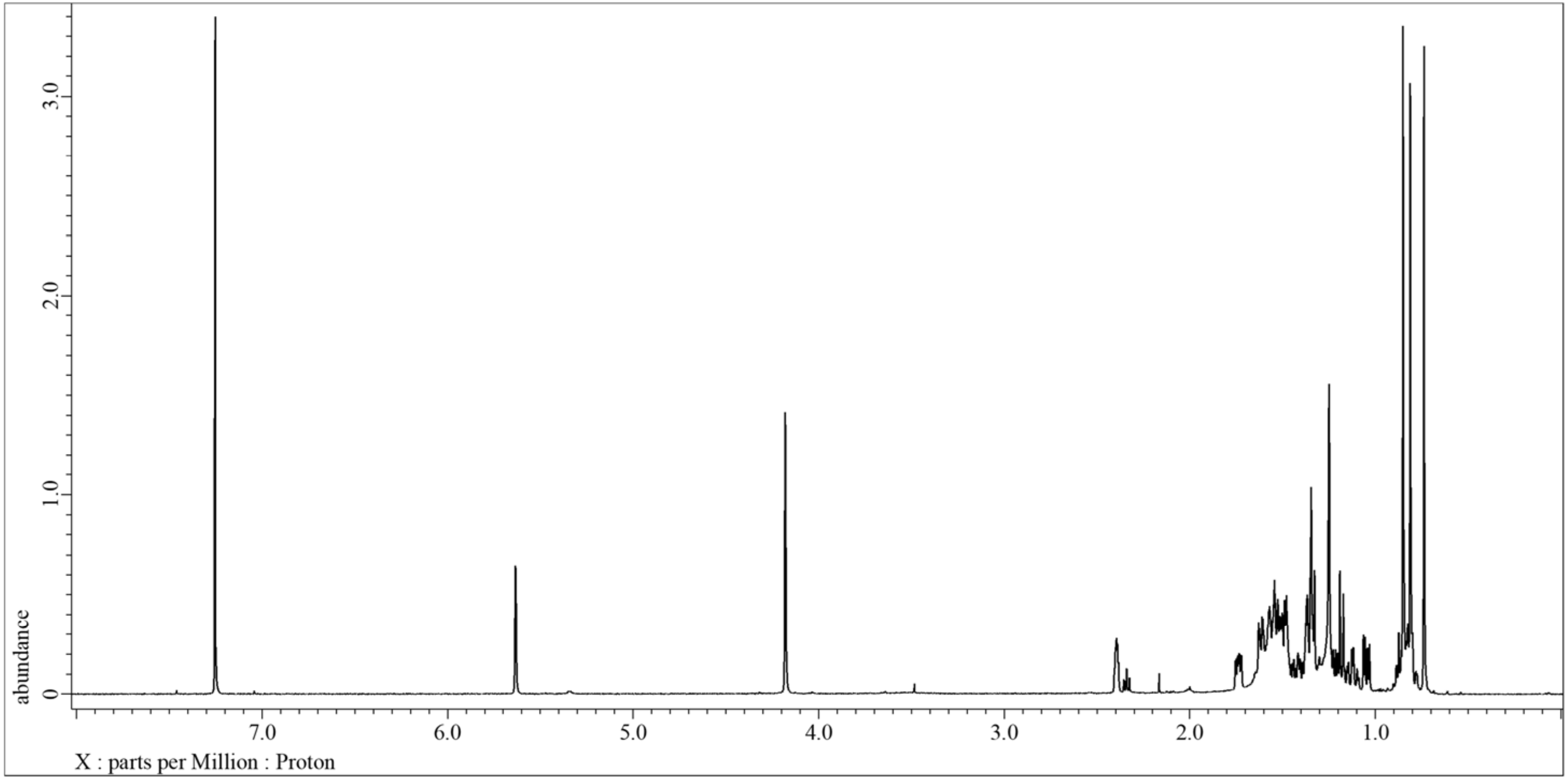

Figure S16. ^{1}H NMR spectrum of **2**.

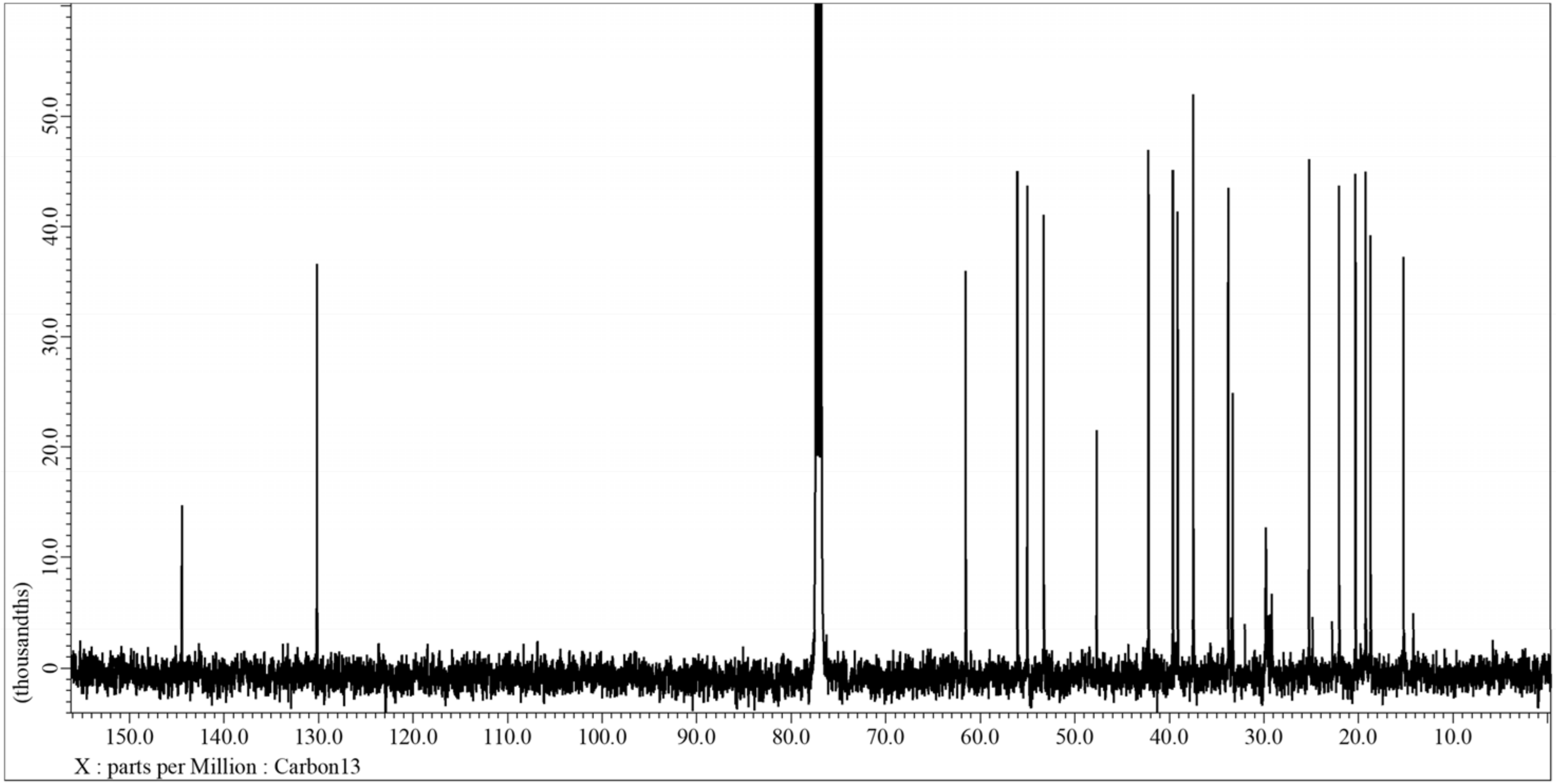

Figure S17. ^{13}C NMR spectrum of **2**.

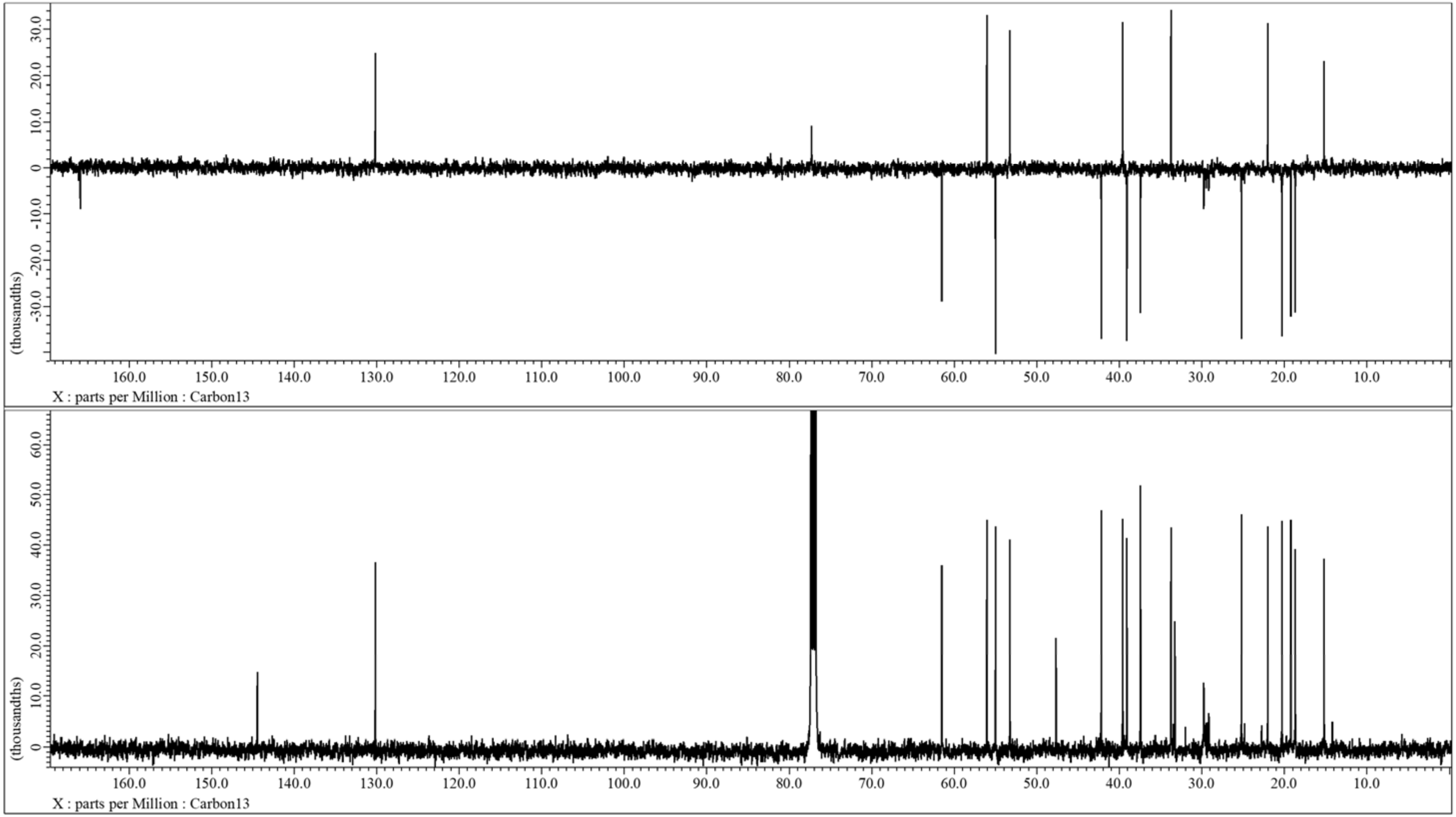

Figure S18. DEPT-135 spectrum of **2**.

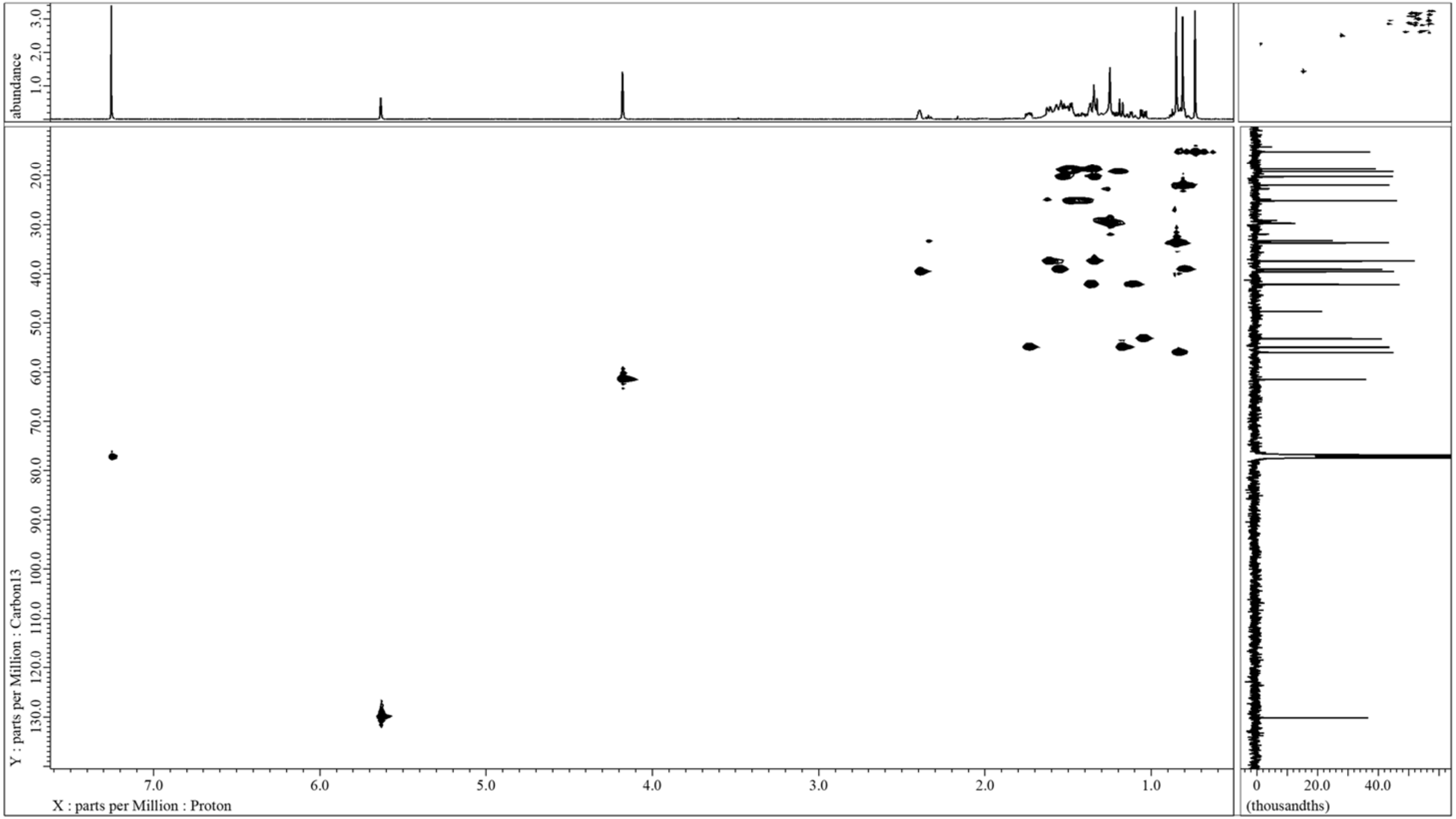

Figure S19. HSQC spectrum of **2**.

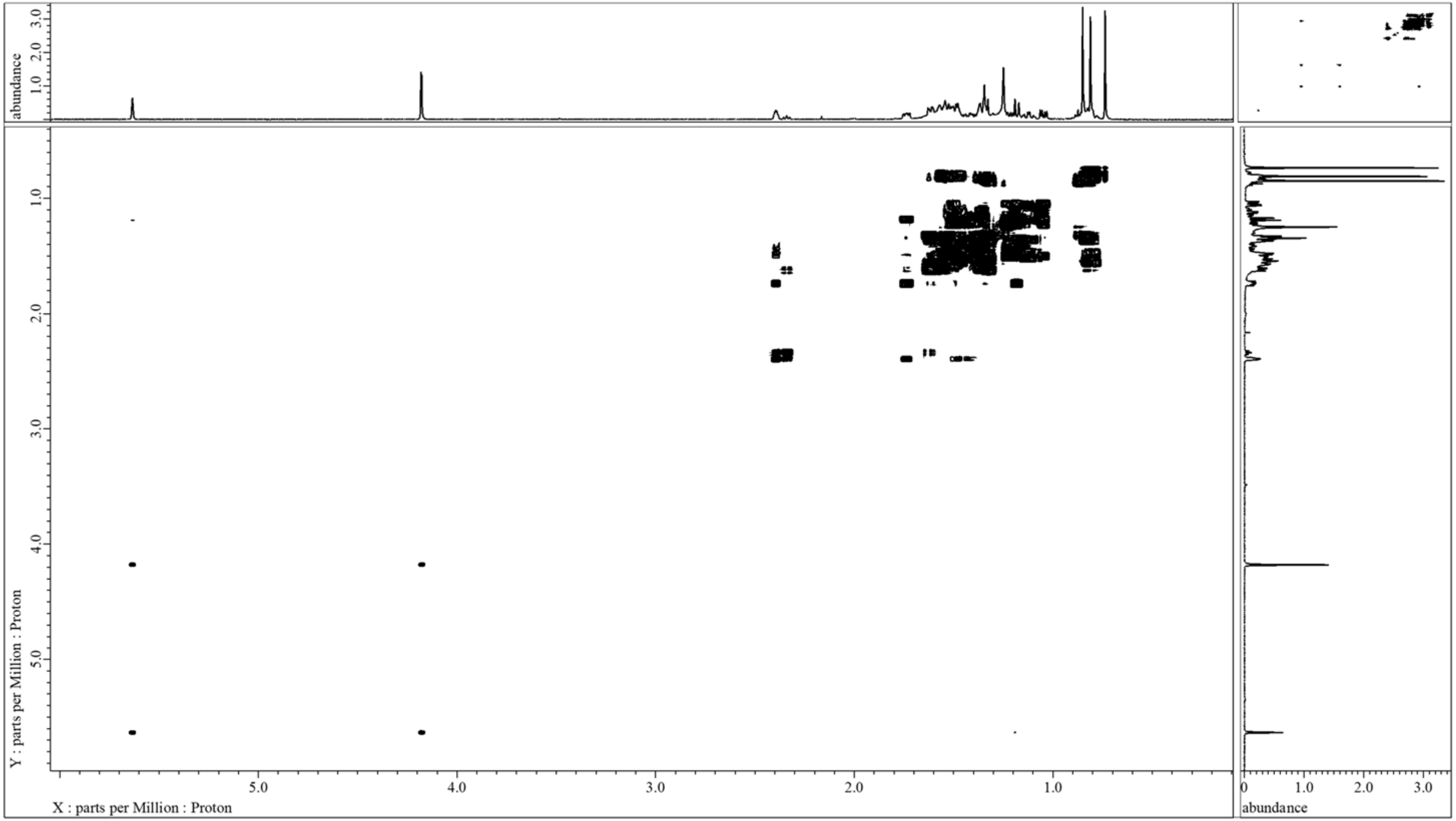

Figure S20. DQF-COSY spectrum of **2**.

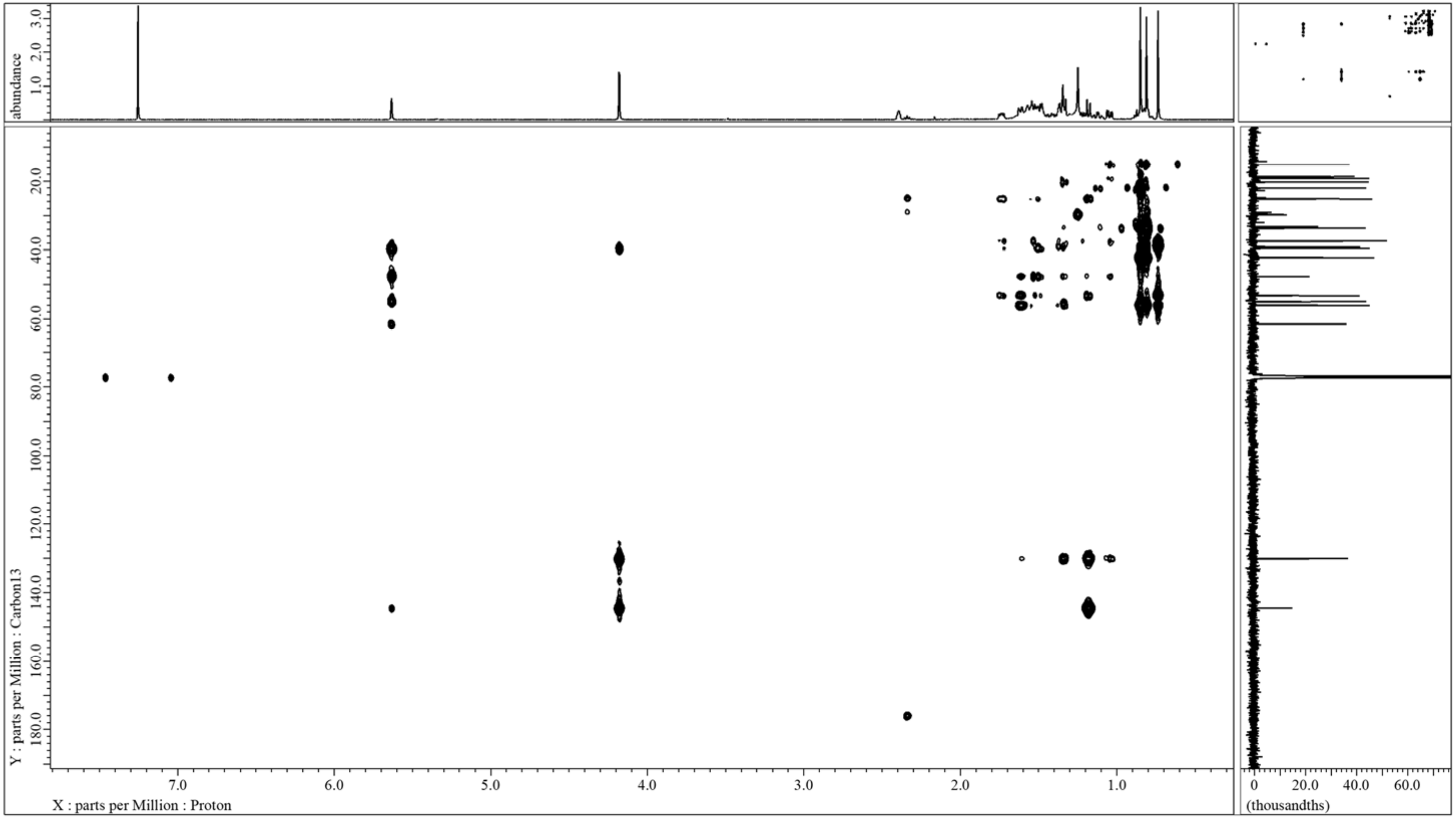

Figure S21. HMBC spectrum of **2**.

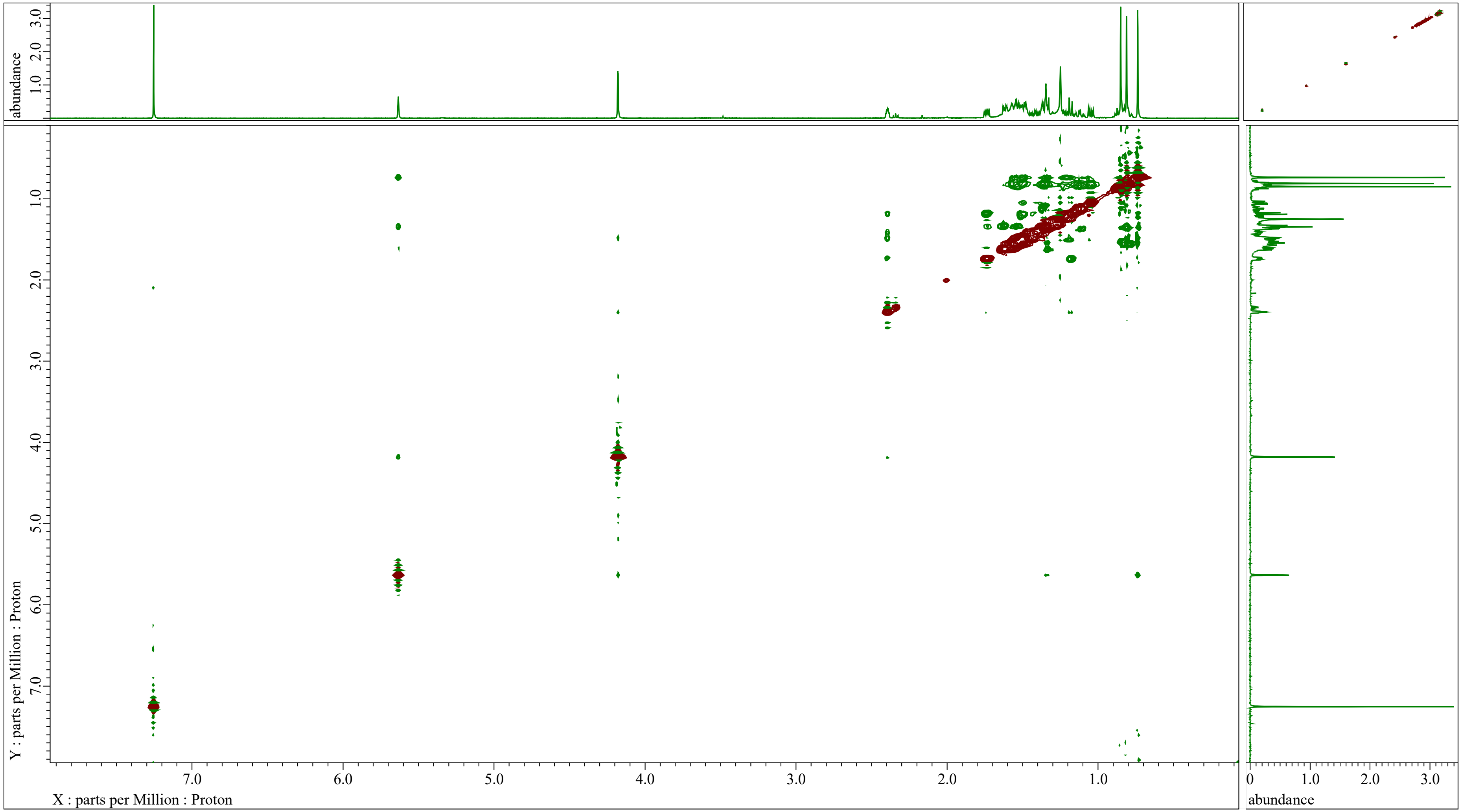

Figure S22. NOESY spectrum of **2**.

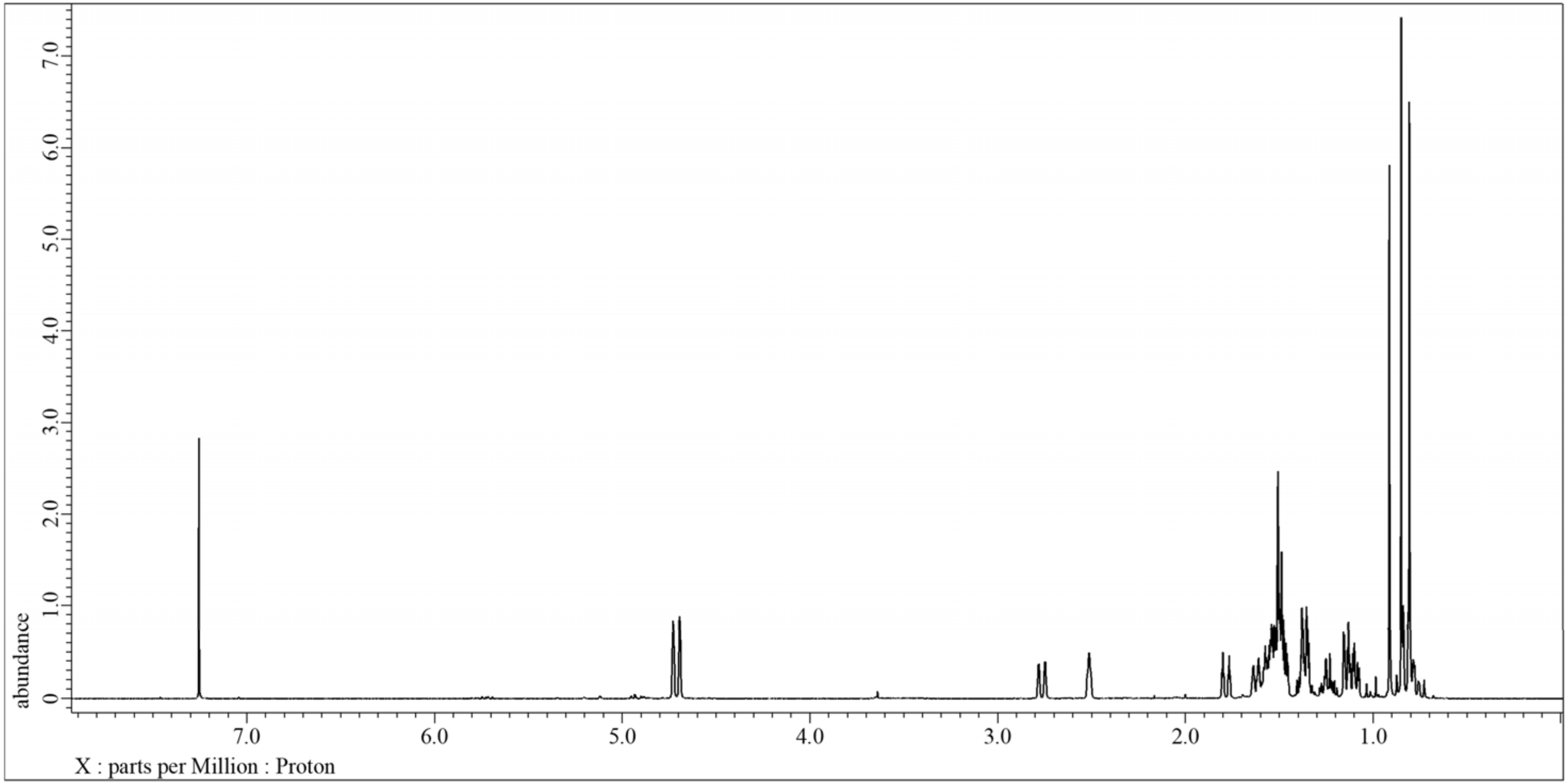

Figure S23. ^{1}H NMR spectrum of **3**.

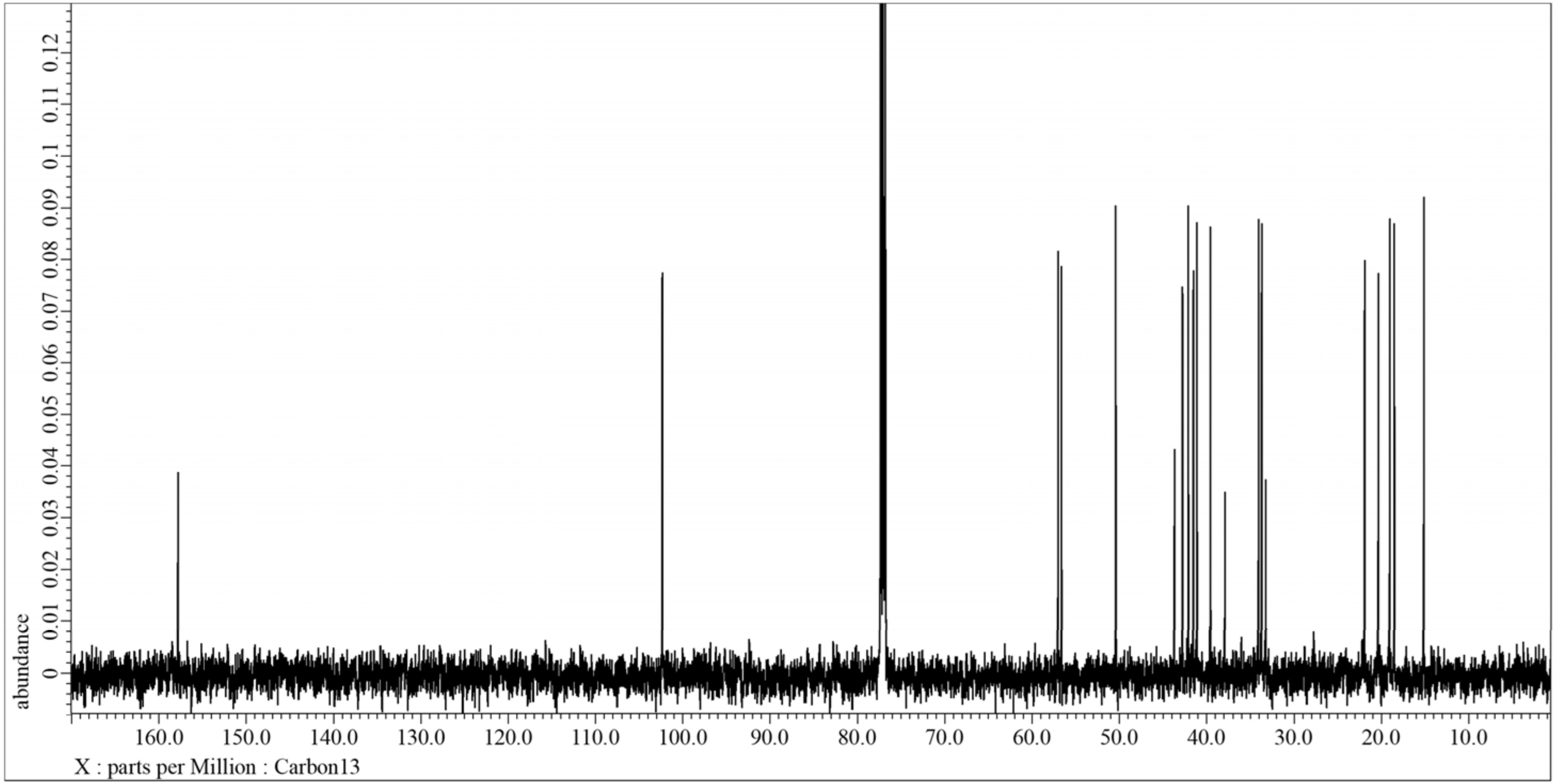

Figure S24. ^{13}C NMR spectrum of **3**.

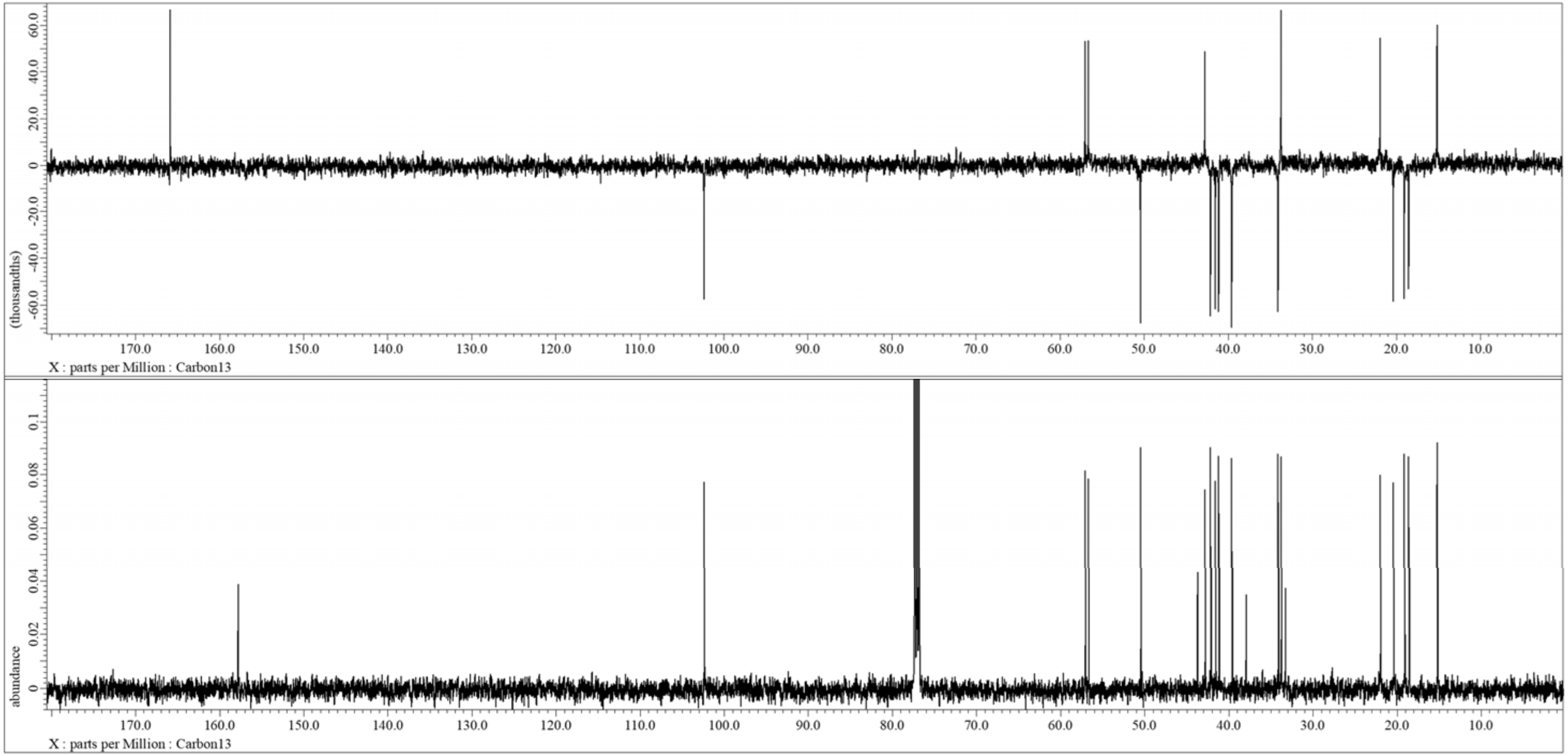

Figure S25. DEPT-135 spectrum of **3**.

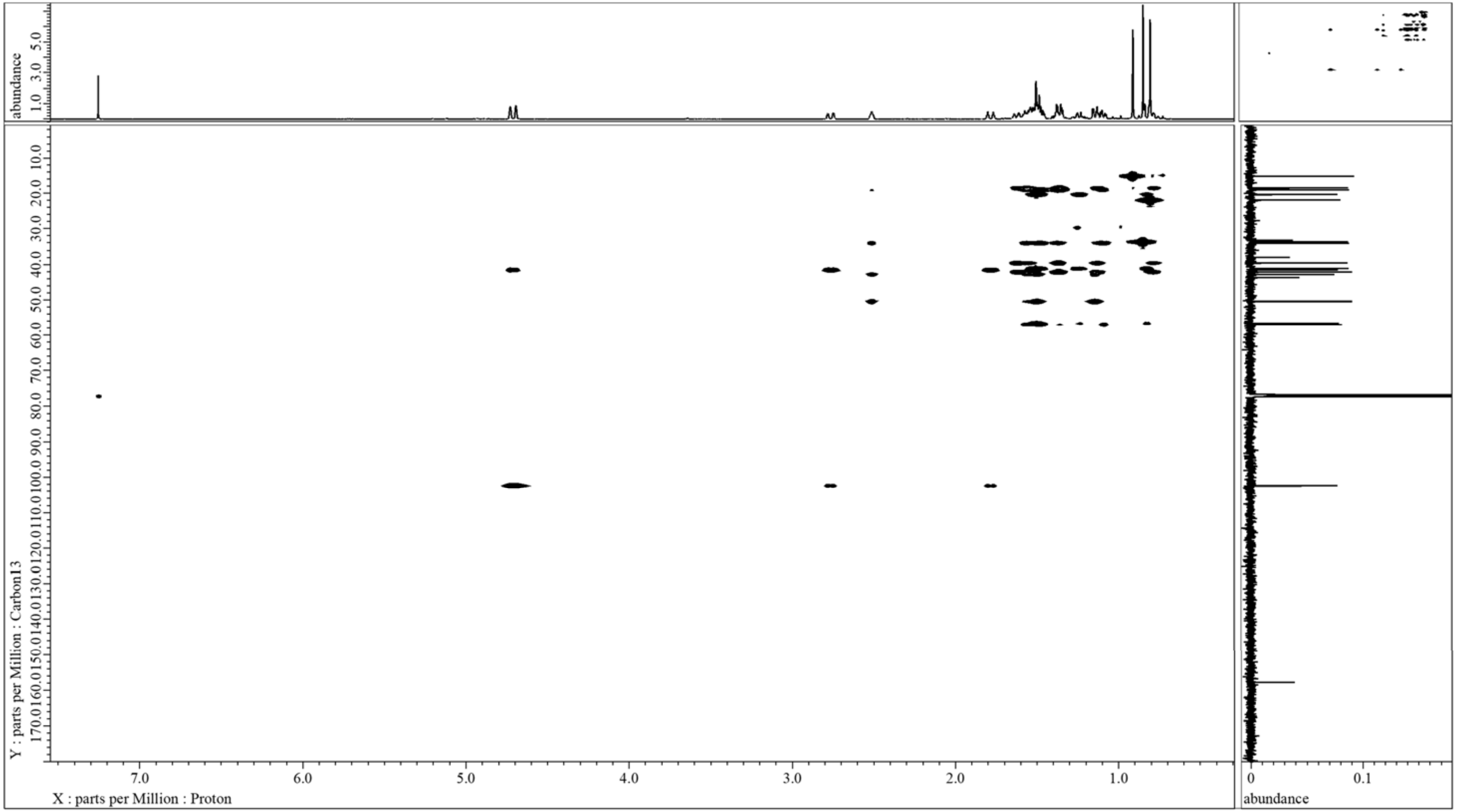

Figure S26. HSQC-TOCSY spectrum of **3**.

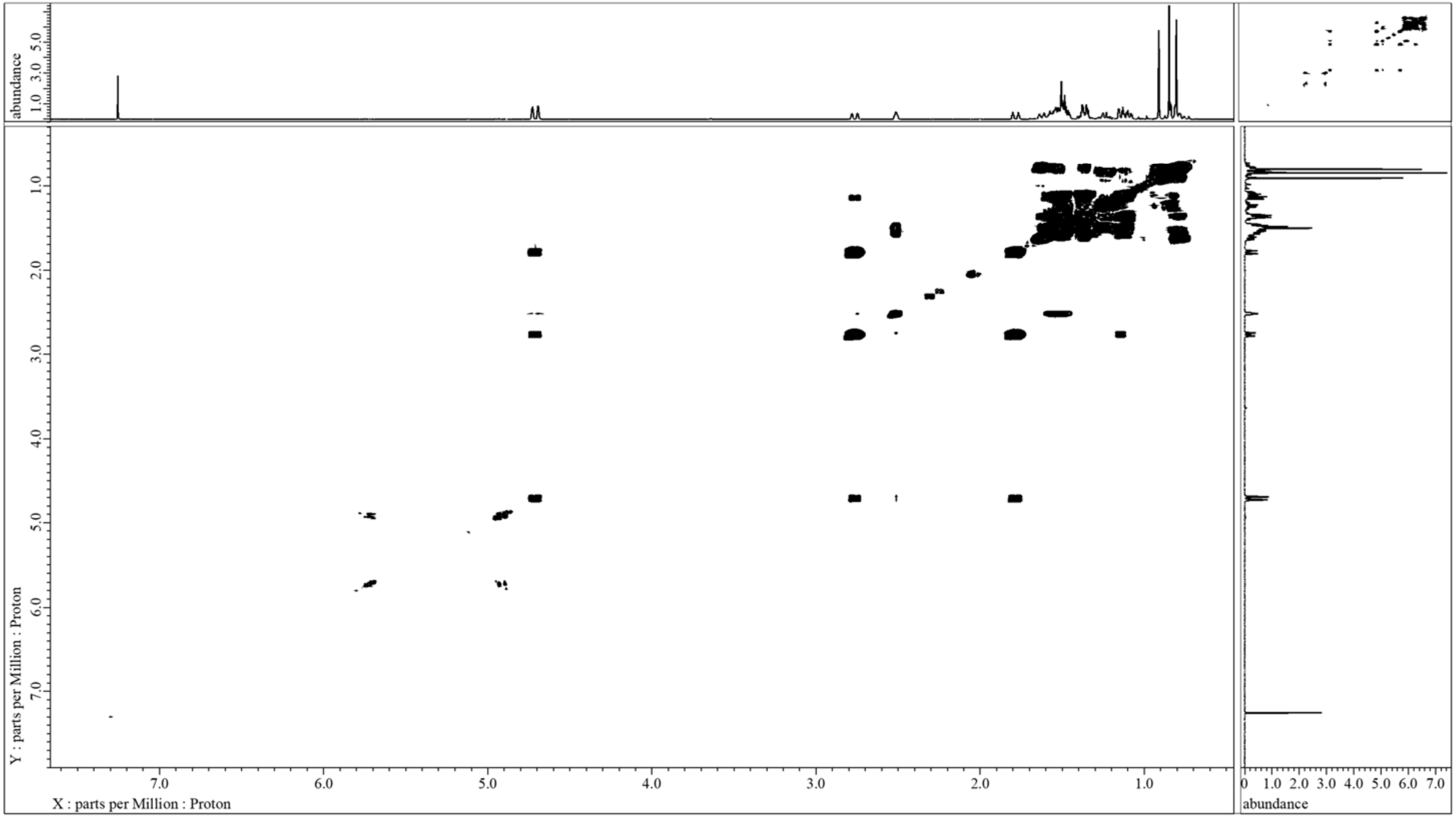

Figure S27. DQF-COSY spectrum of **3**.

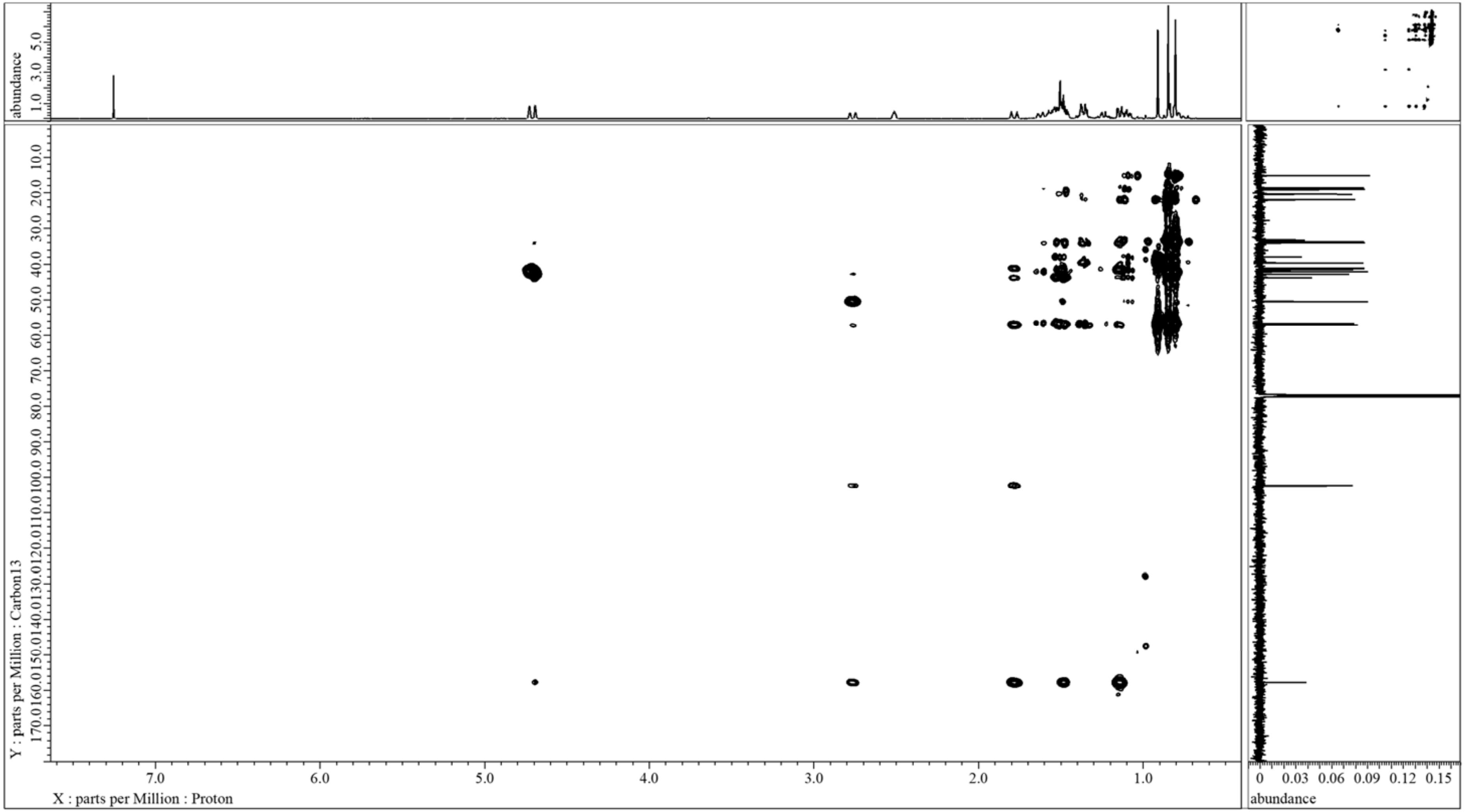

Figure S28. HMBC spectrum of **3**.

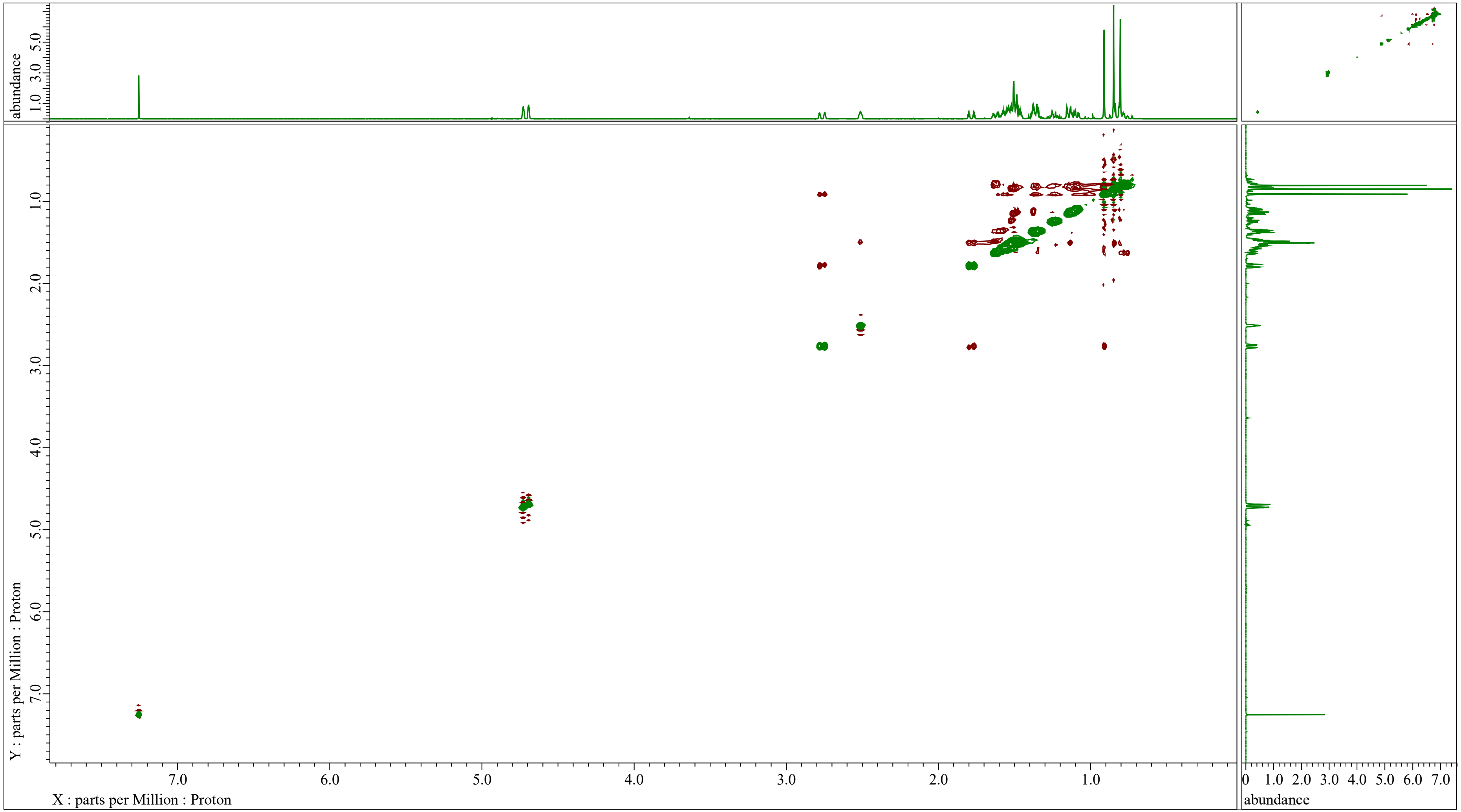

Figure S29. NOESY spectrum of **3**.

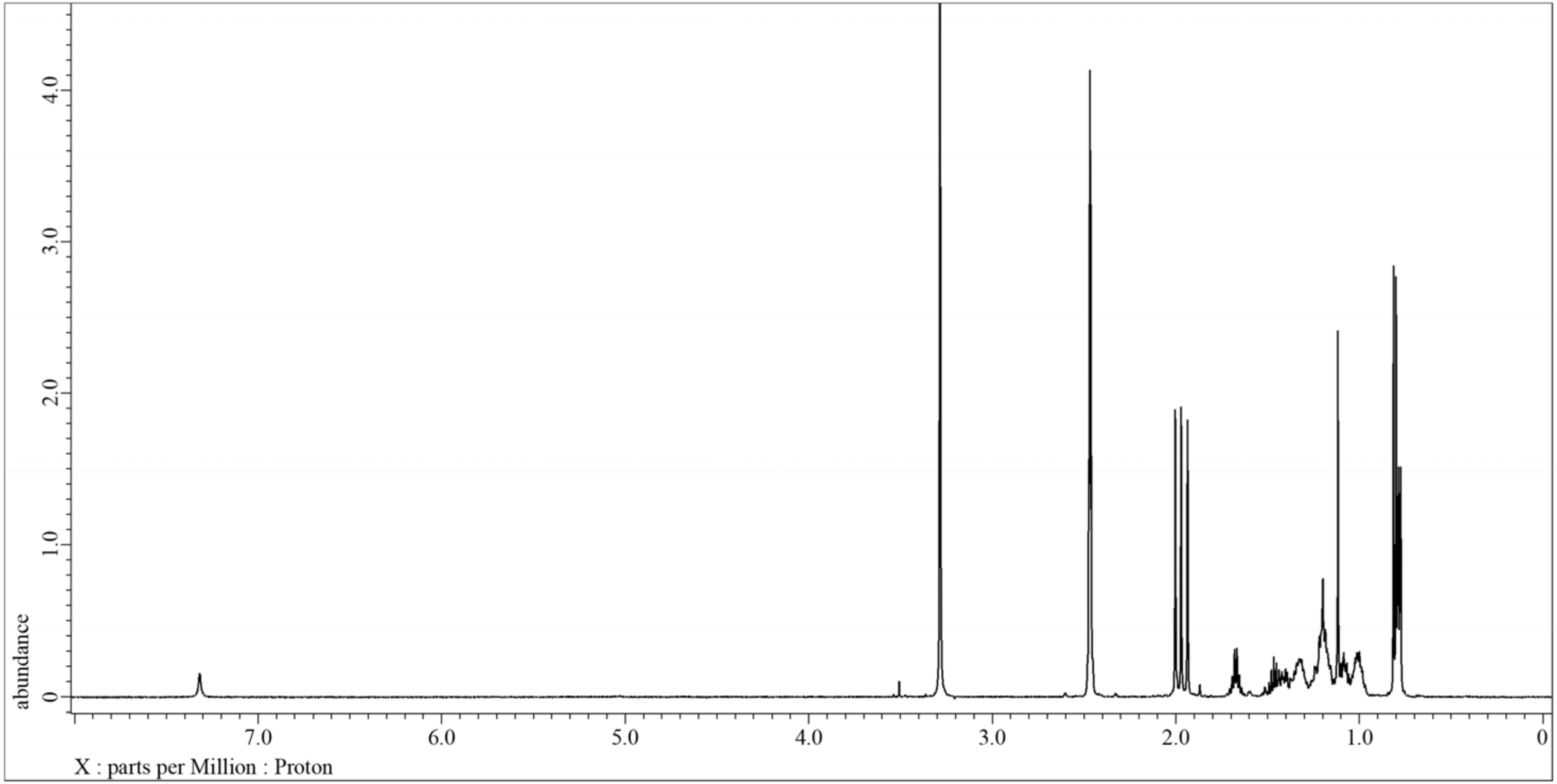

Figure S30. ^{1}H NMR spectrum of **4**.

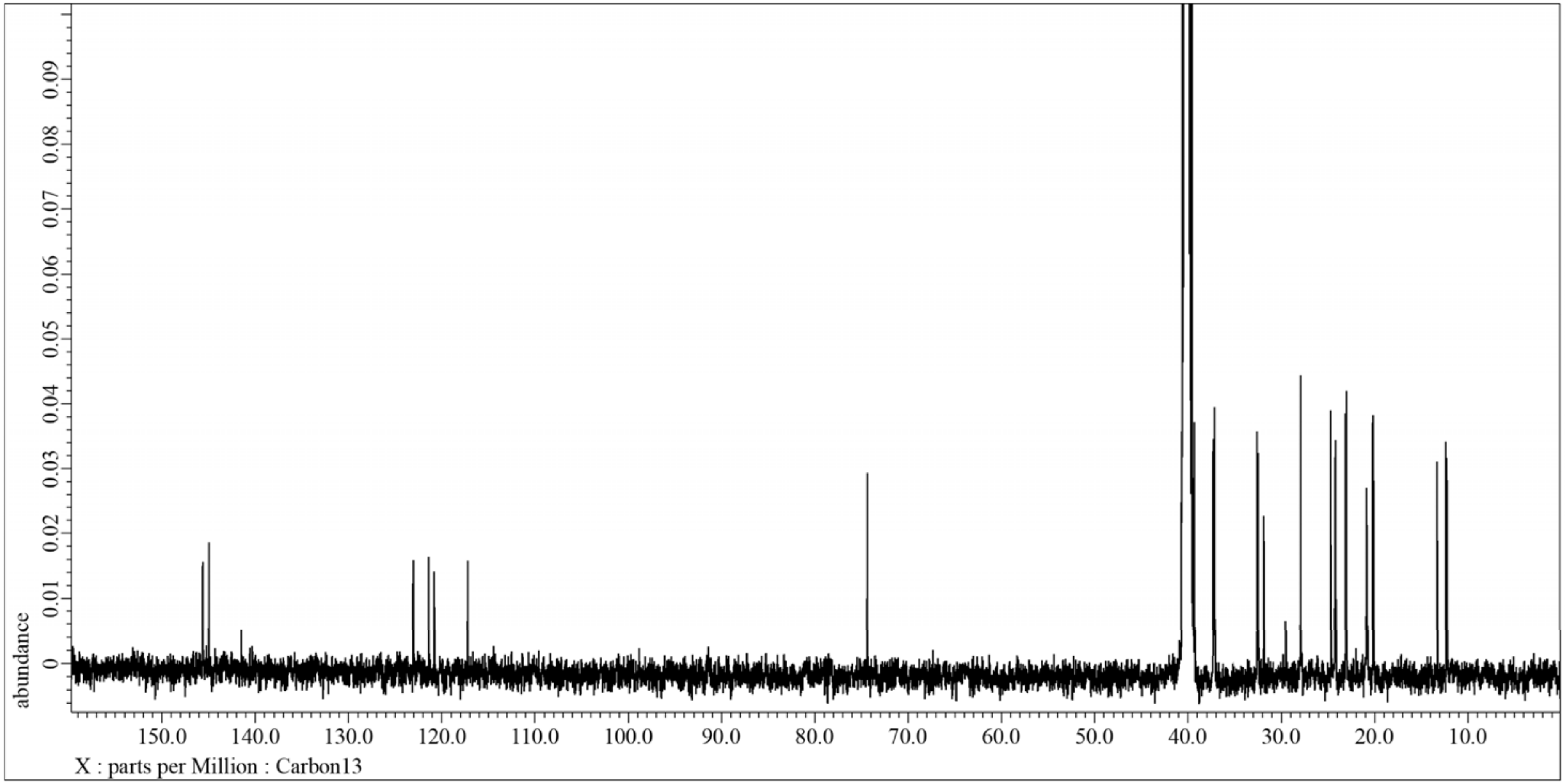

Figure S31. ^{13}C NMR spectrum of **4**.

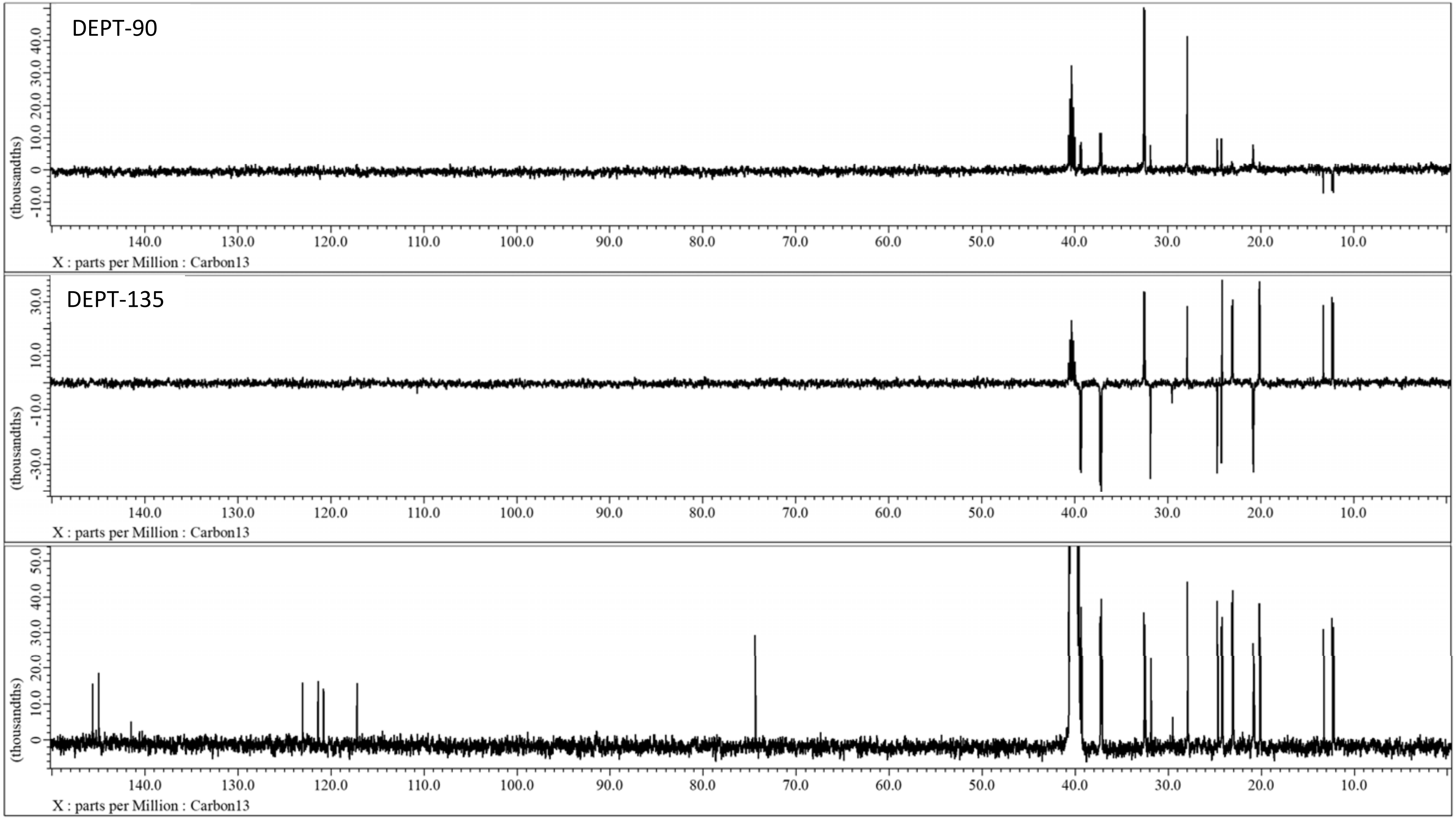

Figure S32. DEPT-90 and DEPT-135 experiments of **4**.

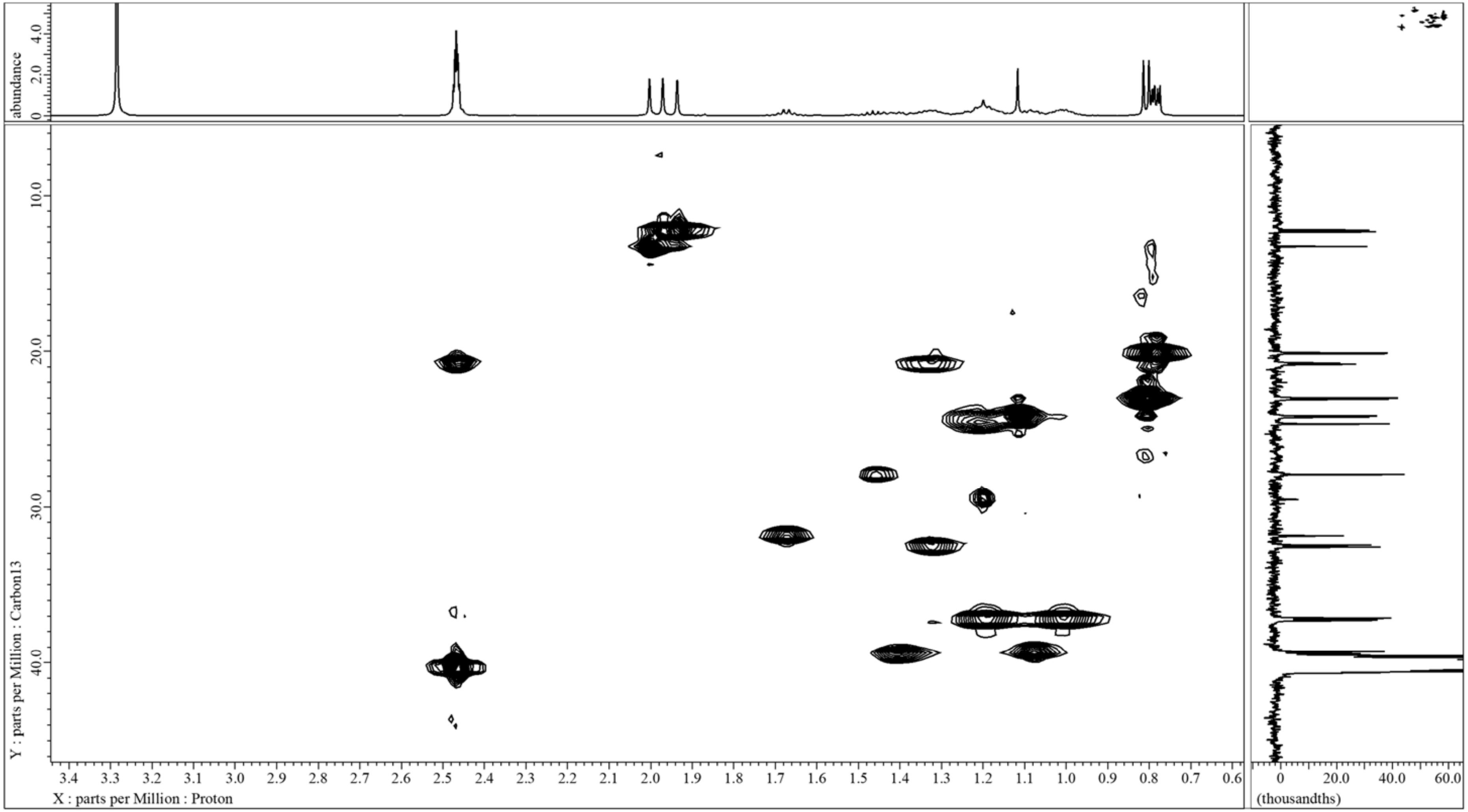

Figure S33. HSQC spectrum of **4**.

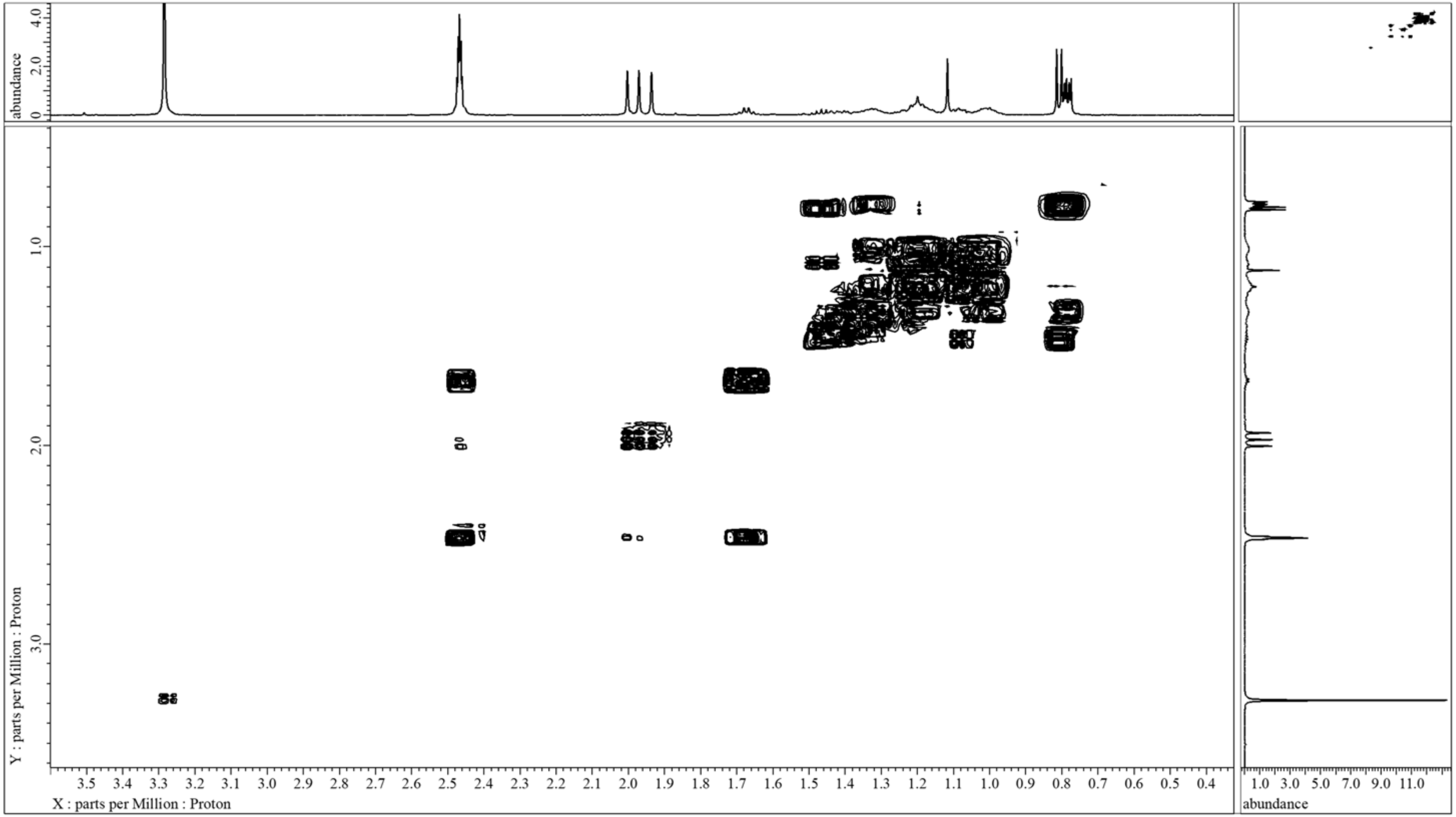

Figure S34. DQF-COSY spectrum of **4**.

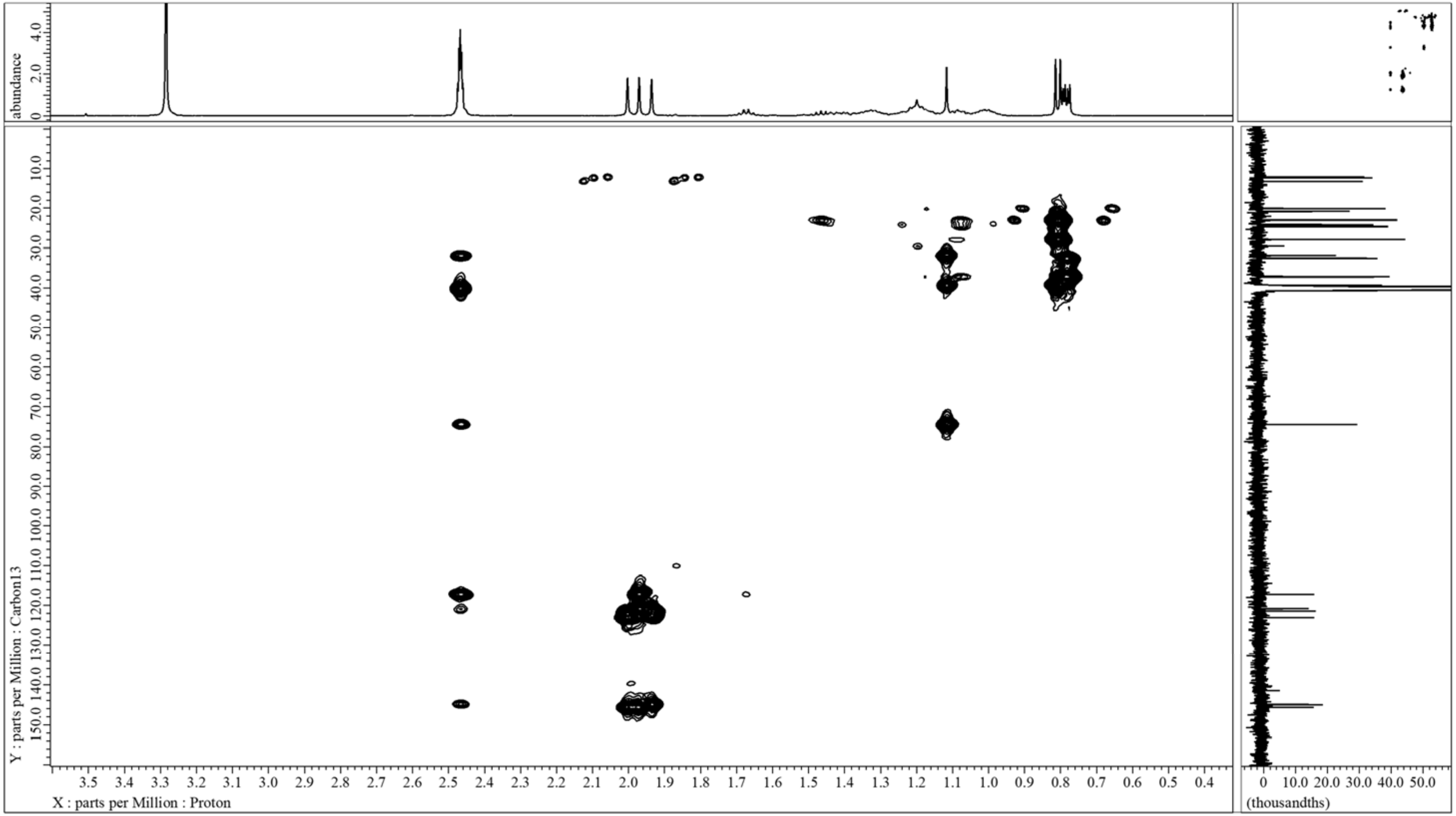

Figure S35. HMBC spectrum of **4**.

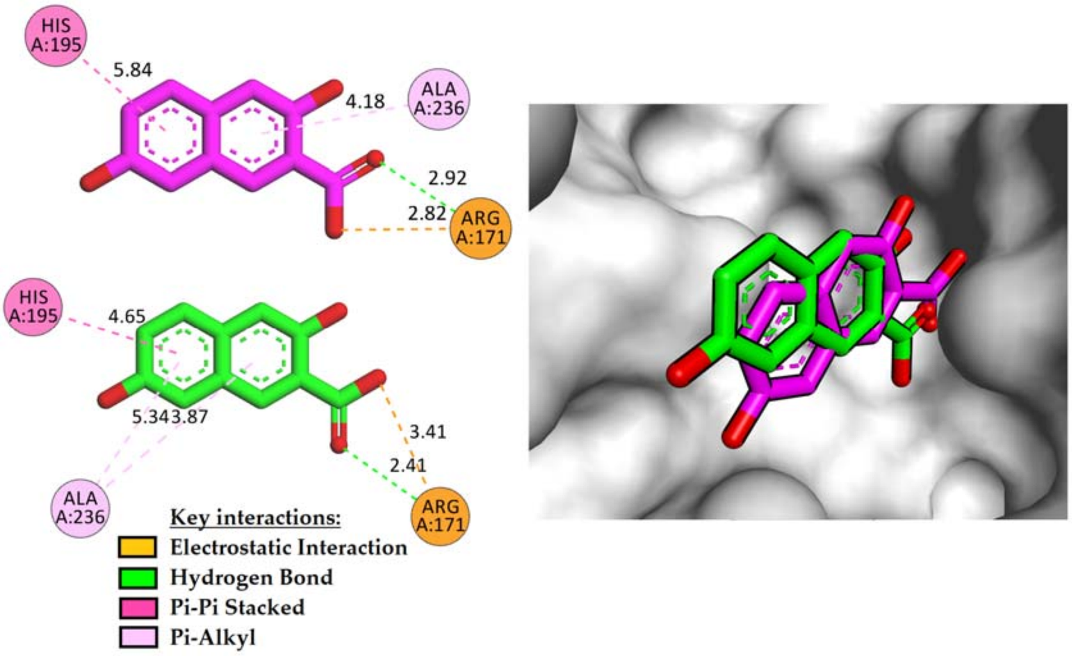

Figure S36. Superimposition of the crystal structure of 3,5-dihydroxy-2-naphthoic acid (C pink and O red) and the docked model (C green and O red) on *Plasmodium falciparum* lactate dehydrogenase crystal structure (PDB ID: 1U5A) with RMSD ~ 2.0 Å and their 2D interactions into the binding site. These models were generated using the Discovery Studio visualizer. The symbol A refers to the amino acids from *Pf*LDH.

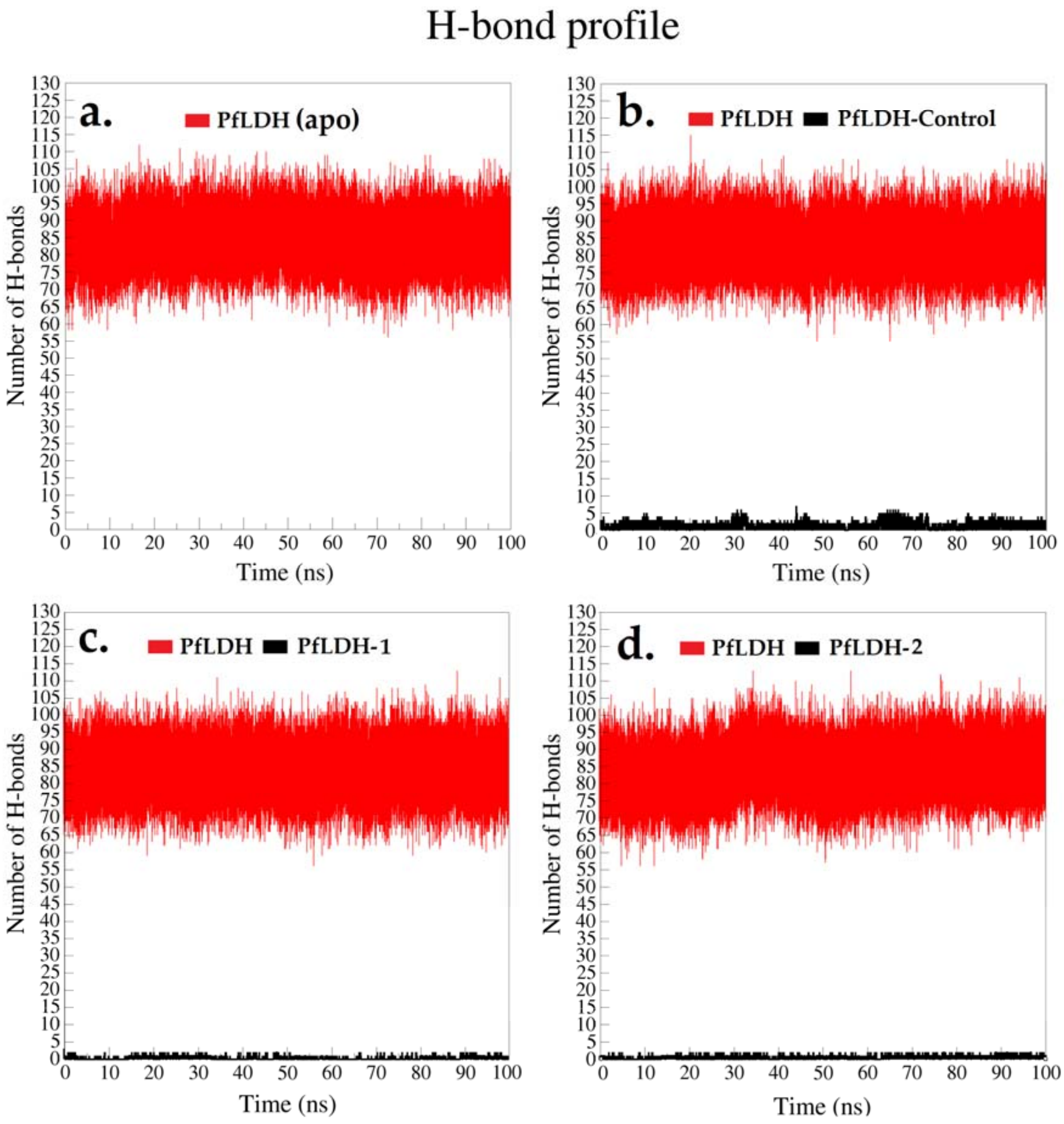

Figure 37. Hydrogen bond profiles obtained from MD simulations (0–100 ns) for all systems: (a) *Pf*LDH-apo, (b) *Pf*LDH- control, (c) *Pf*LDH-**1**, and (d) *Pf*LDH-**2**. The H-bond interactions between enzyme residues are represented in red, while the enzyme-ligand interactions are in black.

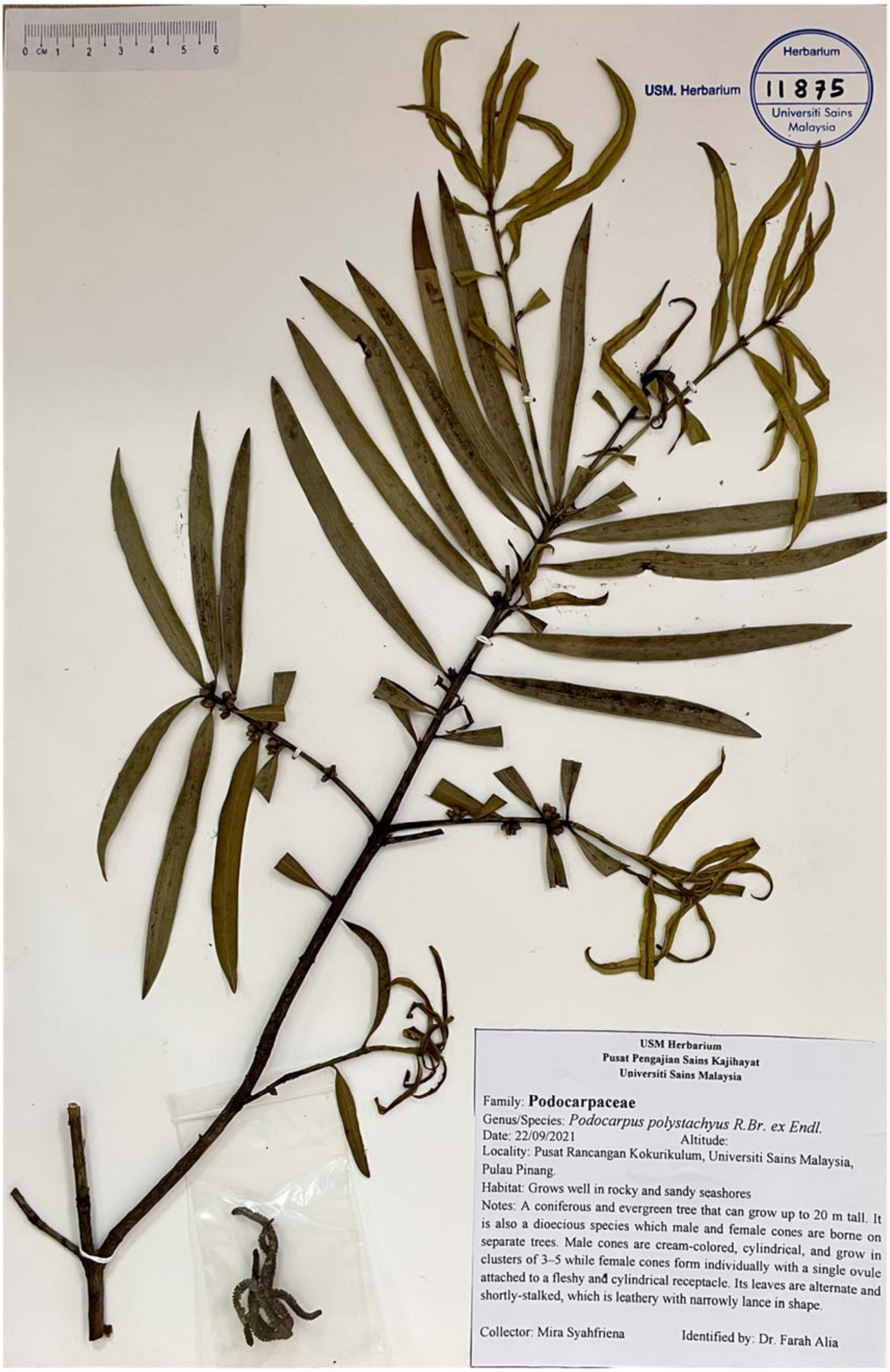

Figure S38. Specimen ID and sample report of *P. polystachyus.*

Table S1. Infection rates of compounds **1** and **2** in comparison to artemisinin as the reference drug.

Compound	Concentration	Average of infection rate (%)	Standard deviation	n=1	n=2	n=3
**1**	0.01	106	10	118	103	98
[µg/ml]	0.03	106	5	111	100	106
	0.1	103	6	98	101	109
	0.3	108	7	116	106	102
	1	87	4	87	83	91
	3	53	8	45	60	54
	10	20	7	27	19	14
	30	14	2	17	13	13
**2**	0.01	107	10	118	103	99
[µg/ml]	0.03	107	4	106	111	103
	0.1	110	9	120	102	108
	0.3	107	3	108	110	103
	1	106	4	104	110	103
	3	99	5	94	99	103
	10	62	10	54	58	73
	30	29	12	43	21	23
Artemisinin	0.1	102	5	96	105	104
[nM]	0.3	95	5	100	92	92
	1	100	5	106	96	99
	3	97	7	90	97	104
	10	65	2	68	64	64
	30	12	1	11	13	12
	100	8	1	8	8	6
	300	6	2	7	4	6

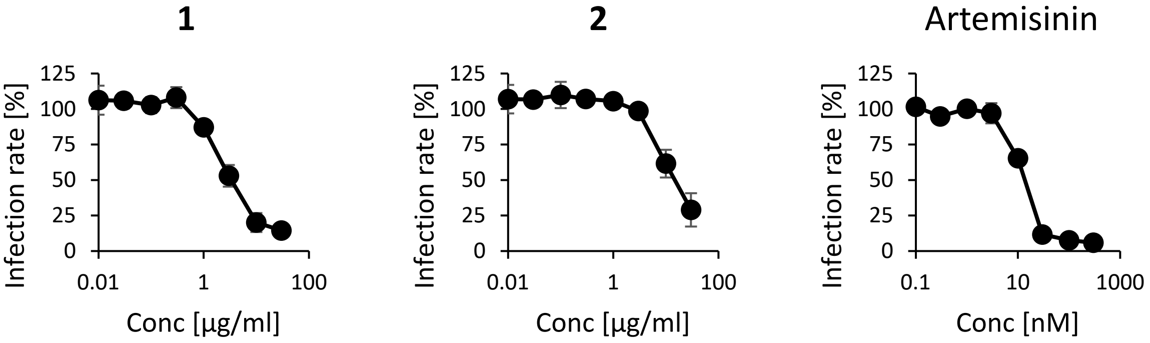

Table S2. IC_{50} of compounds **1** and **2** in comparison to artemisinin as the reference drug.

Compound		Average of IC_{50}	Standard deviation	n=1	n=2	n=3
**1**	[µg/ml]	3.4 (12µM)	0.7	2.6	4.0	3.4
**2**	[µg/ml]	15 (52 µM)	2.0	15	13	17
Artemisinin	[nM]	14	0.4	14	14	13
